# Supplementary material for: Adaptive ionic liquid polymer microwave modulation surface with reprogrammable dielectric properties
Source: Nat Commun. 2026 Jan 8;17:230. doi: 10.1038/s41467-025-68170-w (PMC12783671; doi:10.1038/s41467-025-68170-w)
Supplement: Supplementary file 1 — Supplementary Information [file 41467_2025_68170_MOESM1_ESM.pdf]

Supplementary Information

Adaptive ionic liquid polymer microwave modulation surface with reprogrammable dielectric properties

Qichao Dong[1,2], Zhehui Wang[3], Hanyu Qiu[3,4], Xiaofeng Gong[5], Huying Yan[1], Zengyong Chu[5,6,*], Tao Luo[3,*], Haipeng Lu[1,2,*], Longjiang Deng[1,2].

[1] National Engineering Research Center of Electromagnetic Radiation Control Materials, School of Electronic Science and Engineering, University of Electronic Science and Technology of China; Chengdu, 611731, China.

[2] Key Laboratory of Multispectral Absorbing Materials and Structures of Ministry of Education, School of Electronic Science and Engineering, University of Electronic Science and Technology of China; Chengdu, 611731, China.

[3] Institute of High Performance Computing (IHPC), Agency for Science, Technology and Research (A*STAR), 1 Fusionopolis Way, #16-16 Connexis, Singapore 138632, Republic of Singapore

[4] School of Science and Engineering, The Chinese University of HongKong, Shenzhen, 518172, China

[5] College of Science, National University of Defense Technology, Changsha, 410073 P. R. China

[6] Science and Technology on Advanced Ceramic Fibers and Composites Laboratory, College of Aerospace Science and Engineering, National University of Defense Technology, Changsha, 410073 P. R. China

E-mail addresses:

Zengyong Chu, Email: chuzy@nudt.edu.cn
Tao Luo, Email: luo_tao@a-star.edu.sg(primary); tluo001@e.ntu.edu.sg(alternate)
Haipeng Lu, Email: luhaipeng@uestc.edu.cn

Supplementary Text

The dielectric constant

Typically, the complex permittivity is a dimensionless magnitude related to the permittivity [1]:

$$\varepsilon = \varepsilon' + j\varepsilon'' \text{ (S1)}$$

ε′ is the real part of the permittivity, and ε″ is the imaginary part. Where:

$$\varepsilon' = \frac{\varepsilon}{\varepsilon_0} \text{ (S2)}$$

$$\varepsilon'' = \varepsilon''_p + \varepsilon''_c \text{ (S3)}$$

$$\varepsilon''_c = \frac{\sigma}{\omega\varepsilon_0} \text{ (S4)}$$

ε''_p and ε''_c represent the polarization and conductivity losses of dielectric materials, respectively, where σ is the conductivity and ω = 2πf is the angular frequency.

The purpose of the Havriliak-Negami (H-N) model is to fit and represent the real and imaginary parts of the dielectric constant as functions of frequency, providing a unified description and parameterization of the experimental data [2]:

$$\varepsilon = \varepsilon' - \mathrm{i}\varepsilon'' = -\mathrm{i}\left(\frac{\sigma_0}{\varepsilon_0\omega}\right)^N + \sum_{k=1}^{2}\left[\frac{\Delta\varepsilon_k}{(1+(i\omega\tau_k)^{\alpha_k})^{\beta_k}} + \varepsilon_{\infty,k}\right] \text{ (S5)}$$

Based on the H-N model, ε''_p and ε''_c at different frequencies can be fitted. As shown in Supplementary Fig. 10.

Reflection Loss

According to transmission line theory under metal-backed conditions, the impedance matching coefficient (calculated by Z_{in}/Z_0, where Z_0 = 376.73 Ω) indicates that when Z_{in}/Z_0 is close to 1, electromagnetic wave can enter the IL-P without reflection [3-6]. If the conductivity is too high, as

per Equation S6, the dielectric constant will increase accordingly, causing Z_{in}/Z_0 to deviate from 1, which further leads to reflection of the electromagnetic waves at the air-IL-P interface, making it difficult to achieve excellent microwave absorption performance [7,8]. Therefore, conductivity should be maintained within an optimal range, as both too high and too low are not ideal. As shown in Supplementary Fig. 18, the frequencies corresponding to the dashed lines reveal the ideal impedance matching characteristics for different thicknesses.

$$Z_{in} = Z_0\sqrt{\frac{\mu_r}{\varepsilon_r}}tanh\left[j\frac{2\pi fd}{c}\sqrt{\mu_r\varepsilon_r}\right] \quad \text{(S6)}$$

The attenuation constant (α), used to evaluate the loss capability of electromagnetic waves in IL-P, is as follows[1,8]:

$$\alpha = \frac{\sqrt{2}\pi f}{c} \times \sqrt{(\mu''\varepsilon'' - \mu'\varepsilon') + \sqrt{(\mu''\varepsilon'' - \mu'\varepsilon')^2 + (\mu''\varepsilon' + \mu'\varepsilon'')^2}} \quad \text{(S7)}$$

The reflection loss (RL) at a specific frequency is used to evaluate the microwave absorption, and the formula is as follows[1,8]:

$$RL(dB) = 20log_{10}\frac{|Z_{in}-Z_0|}{|Z_{in}+Z_0|} \quad \text{(S8)}$$

In terms of impedance matching coefficients ($Z = Z' + jZ''$, calculated by Z_{in}/Z_0, Z_0= 376.73031 Ω), when Z is close to 1 ($Z' = 1$ and $Z'' = 0$), the EMWs can enter the absorber without reflection.

Far-field Imaging

The far-field distance refers to the distance from a radiation source to the receiving point, where the electromagnetic field of the source can be considered as a plane wave (i.e., the electric and magnetic field wavefronts approach a plane, and the wavelength variation is minimal). In antenna theory, the far-field distance is a very important concept, as shown in Supplementary Fig. 20.

$$d_f = \frac{2D^2}{\lambda} \quad \text{(S9)}$$

d_f is the far-field distance, D is the maximum size of the sample, and λ is the wavelength of the electromagnetic wave.

Radar Cross Section

Radar Cross Section (RCS) is measured in dB and serves as a crucial metric for assessing an object's visibility to radar systems, with higher values indicating enhanced detectability. The RCS values can be described as follows [1,8,9]:

$$RCS(dB) = \lim_{r \to \infty} 4\pi r^2 \frac{|E_s|^2}{|E_i|^2} \quad \text{(S10)}$$

Supplementary Figure

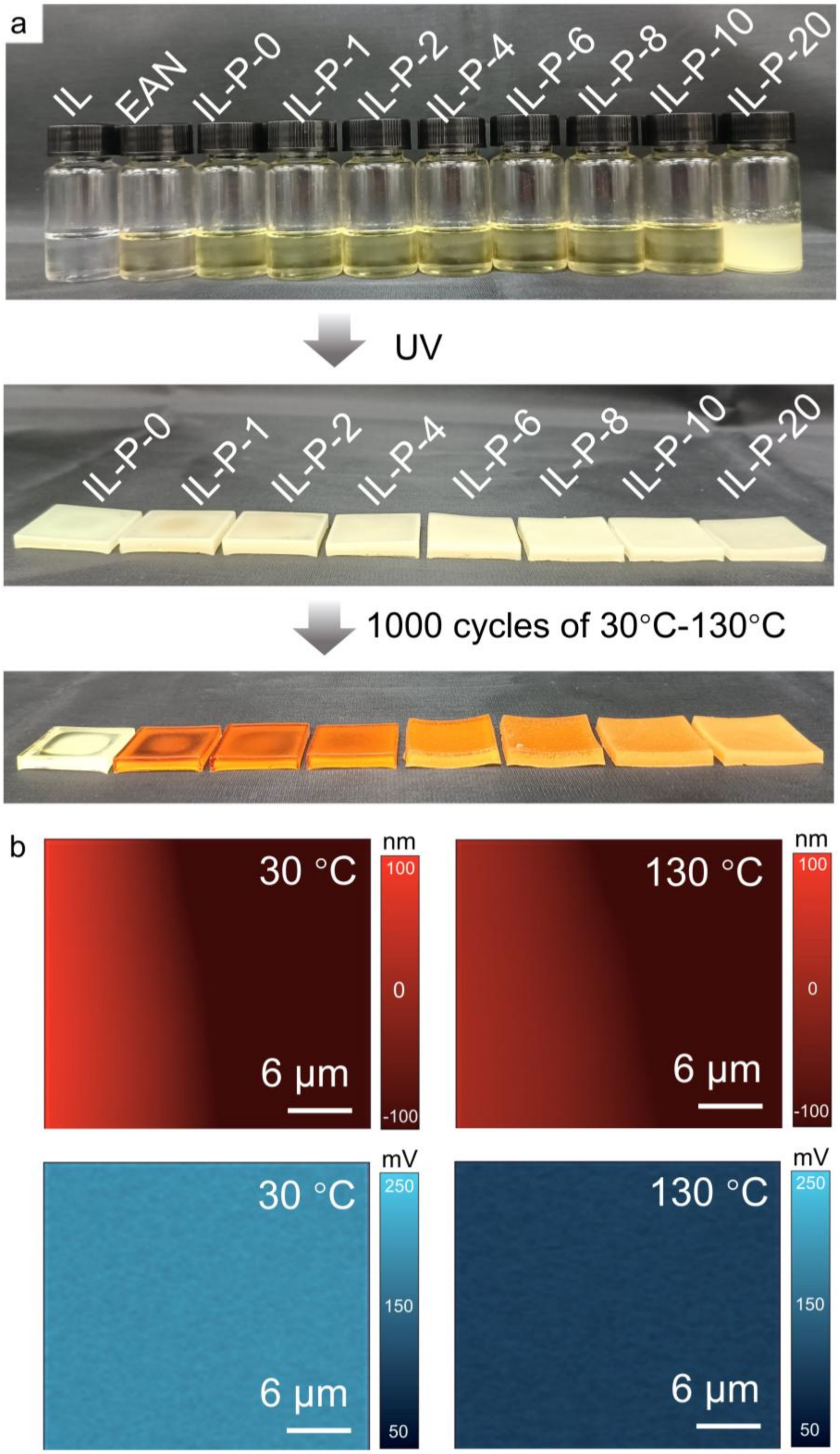

Supplementary Fig. 1 | Morphology of IL-P. a Photograph of the IL-P preparation process. **b** KPFM results of the IL-P film on a silicon substrate in the 800 μm × 800 μm area, with the top image showing the morphology and the bottom image showing the surface potential.

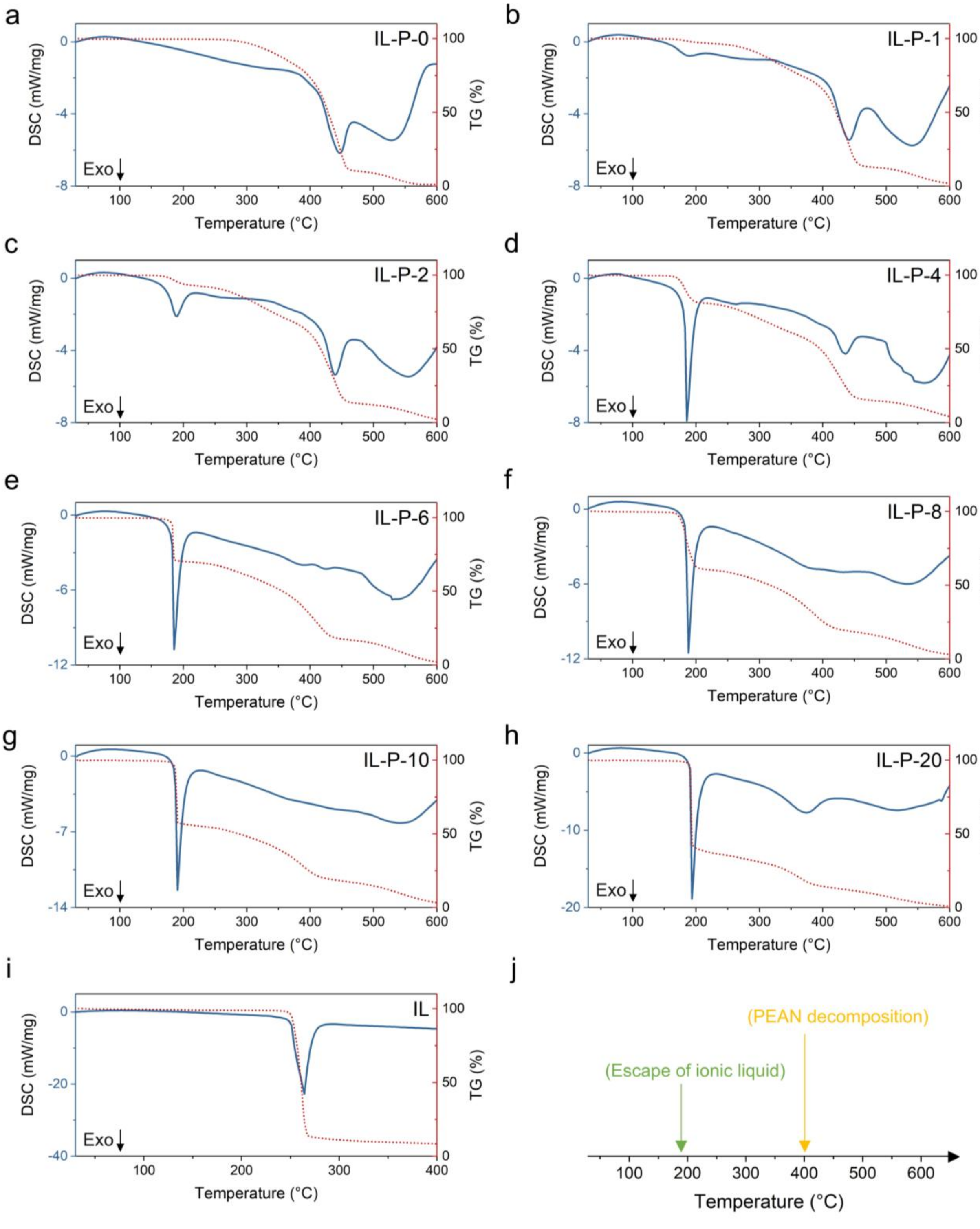

Supplementary Fig. 2 | Thermogravimetric and differential scanning calorimetry (TG-DSC). **a-h** IL-P. **i** IL. **j** Decomposition schematic of IL-P.

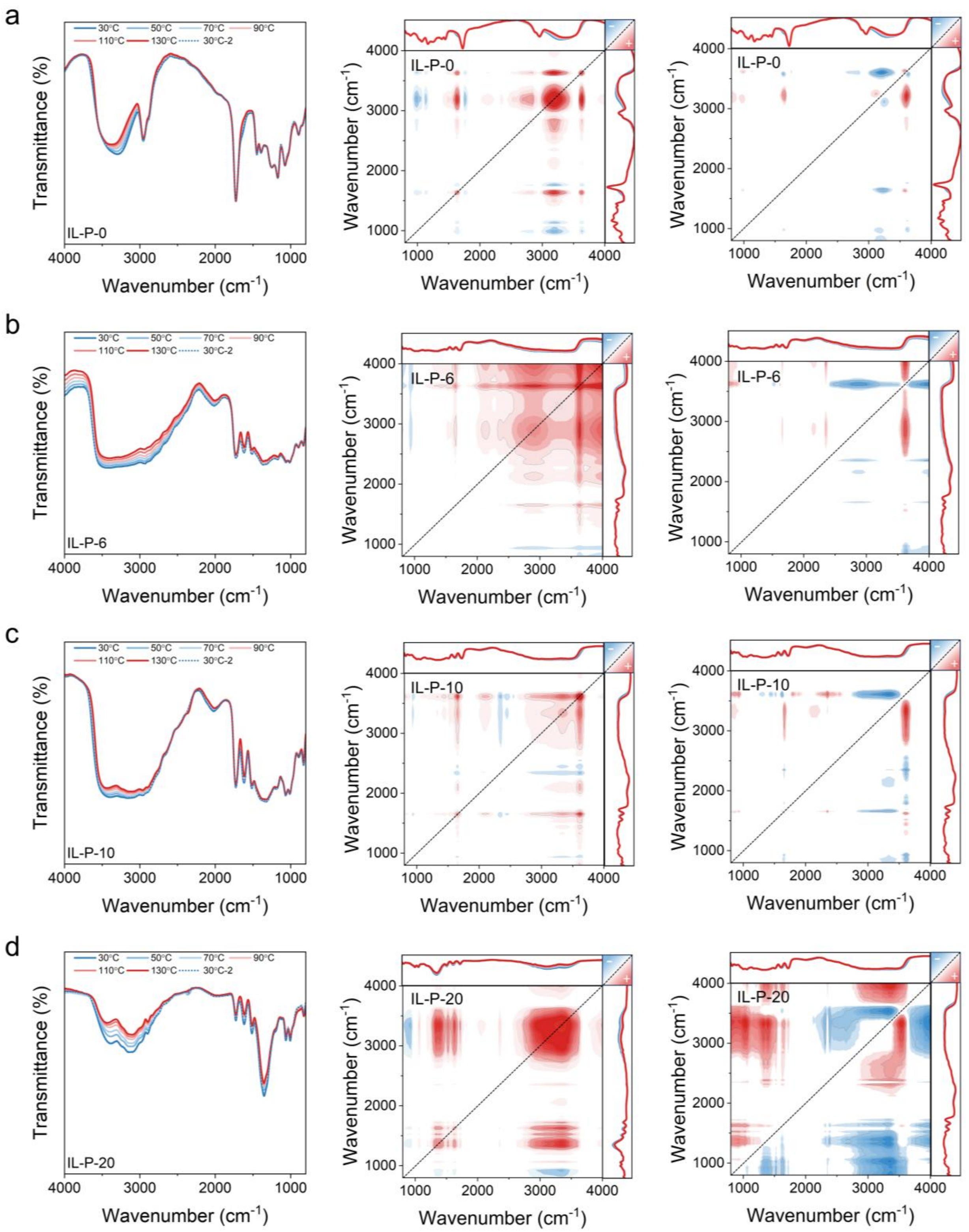

Supplementary Fig. 3 | In situ variable-temperature Fourier-transform infrared (FTIR), 2D synchronous and asynchronous spectra. a IL-P-0. **b** IL-P-6. **c** IL-P-10. **d** IL-P-20.

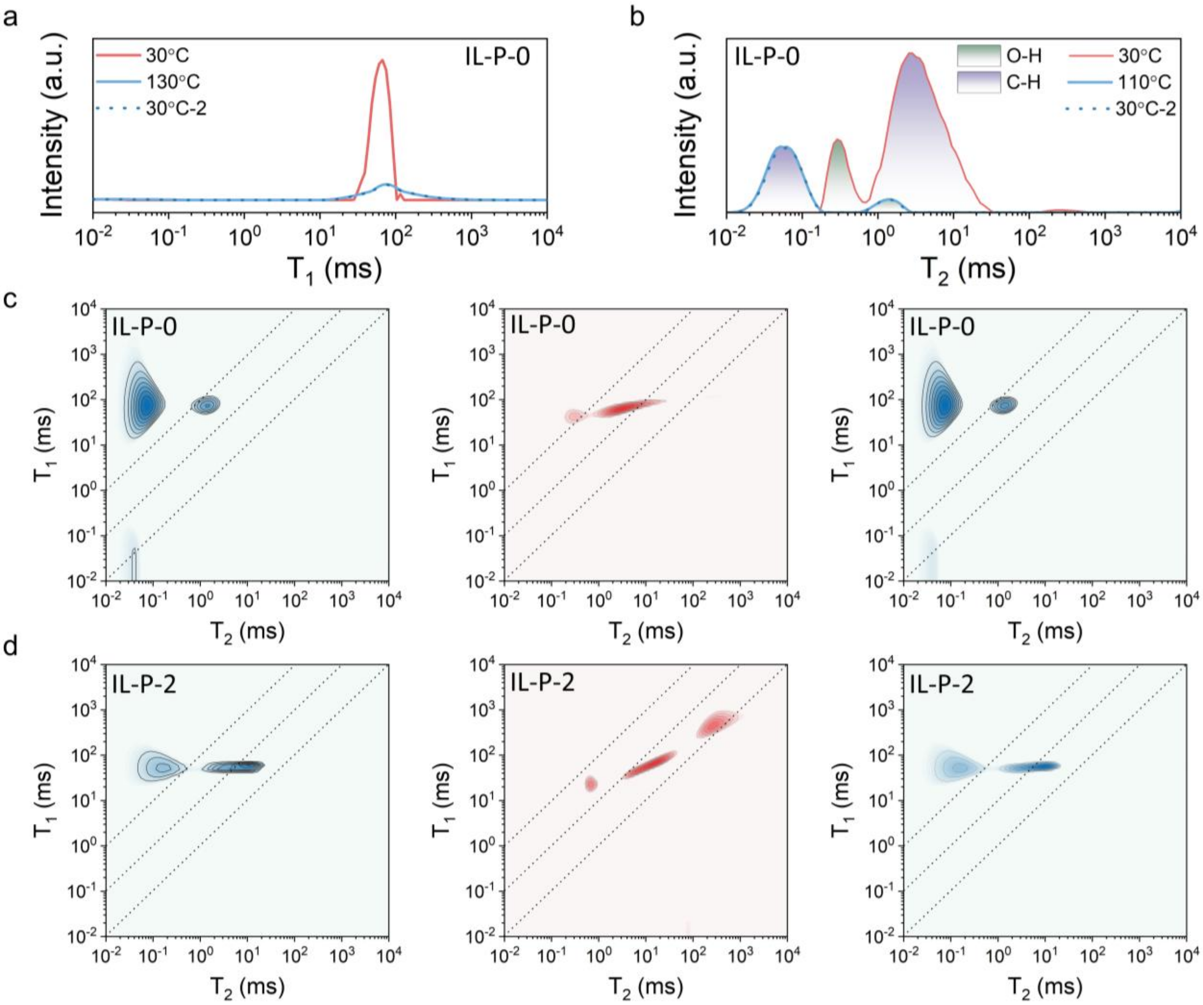

Supplementary Fig. 4 | In situ variable-temperature ¹H low-field NMR. a, b T_1 and T_2 spectra of IL-P-0. **c, d** Variable-temperature ¹H low-field 2D NMR of IL-P-0 and IL-P-2, with measurements taken at 30°C, heated to 130°C, and then cooled back to 30°C from left to right.

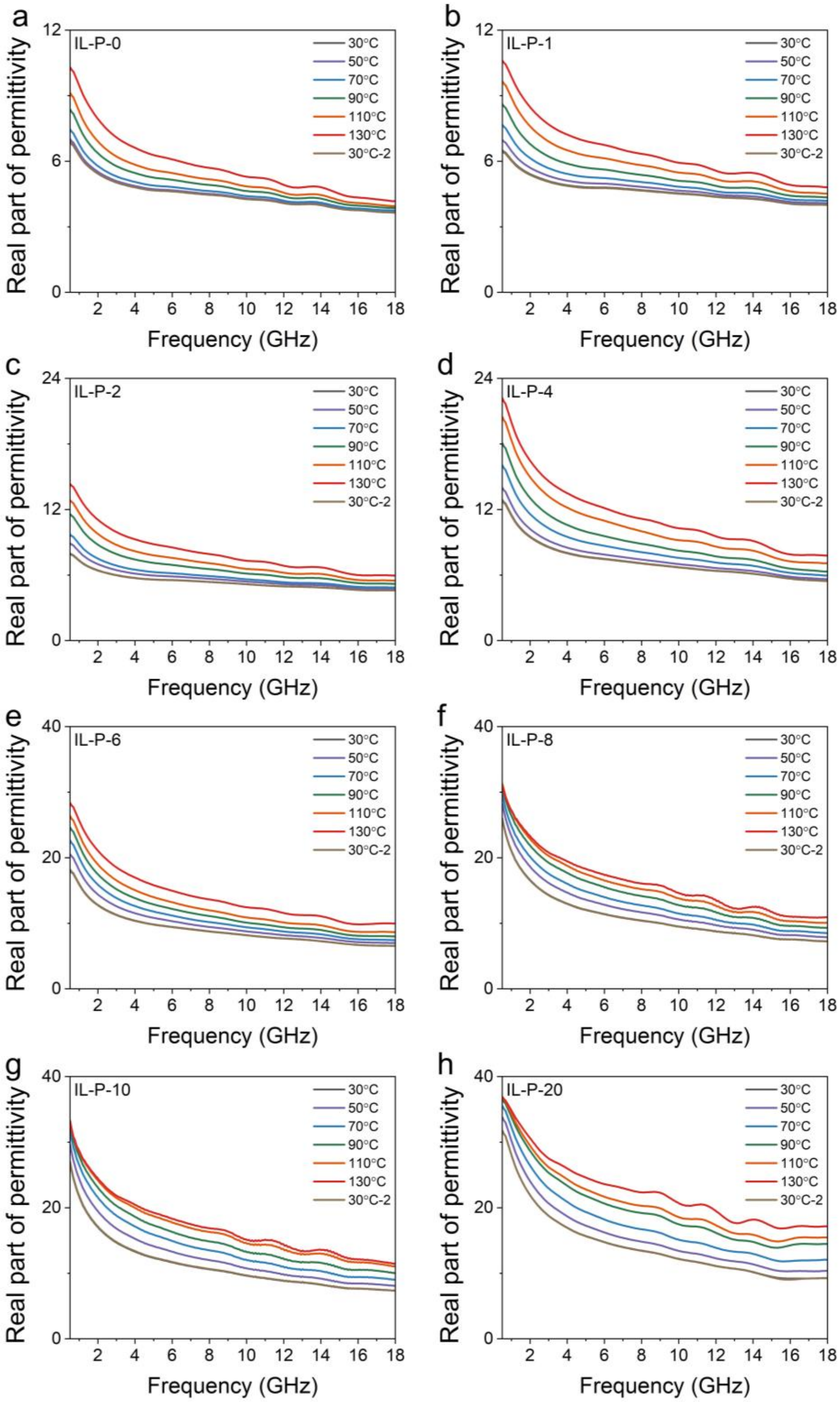

Supplementary Fig. 5 | Real part of permittivity of IL-P in the 0.5–18 GHz under temperature stimulation. **a** IL-P-0. **b** IL-P-1. **c** IL-P-2. **d** IL-P-4. **e** IL-P-6. **f** IL-P-8. **g** IL-P-10. **h** IL-P-20.

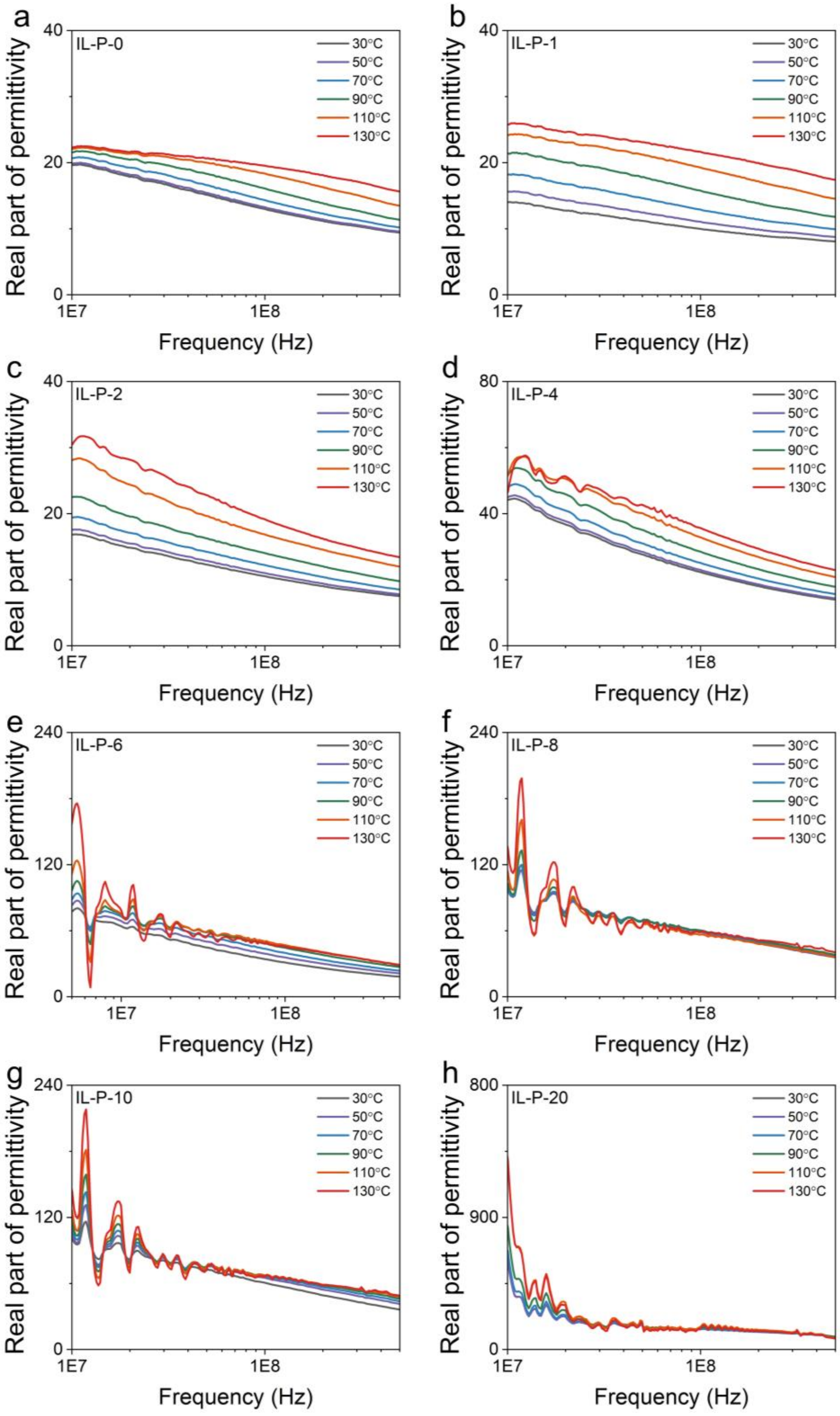

Supplementary Fig. 6 | Real part of permittivity of IL-P in the 10^7-5 × 10^8 Hz under temperature stimulation. **a** IL-P-0. **b** IL-P-1. **c** IL-P-2. **d** IL-P-4. **e** IL-P-6. **f** IL-P-8. **g** IL-P-10. **h** IL-P-20.

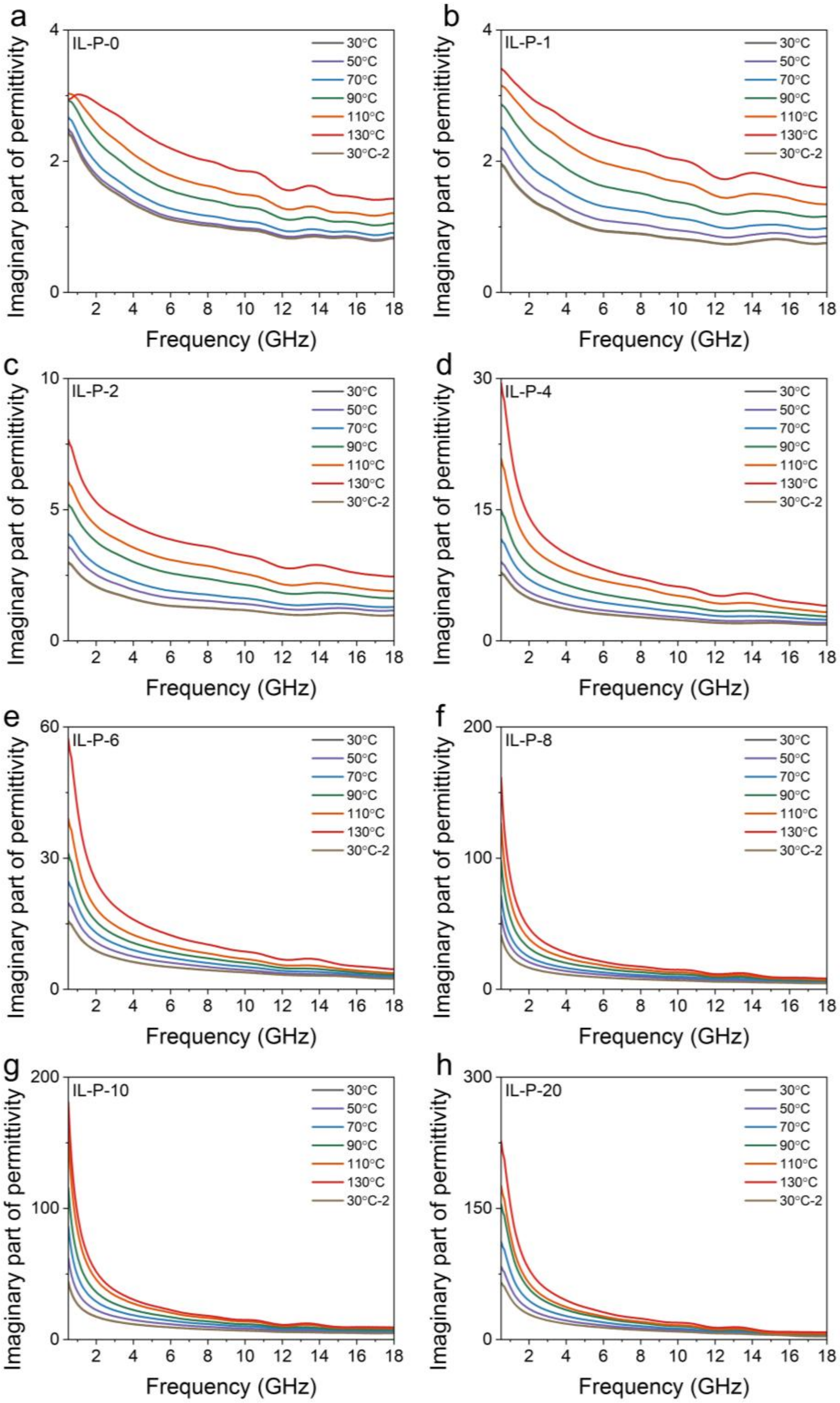

Supplementary Fig. 7 | Imaginary part of permittivity of IL-P in the 0.5–18 GHz under temperature stimulation. **a** IL-P-0. **b** IL-P-1. **c** IL-P-2. **d** IL-P-4. **e** IL-P-6. **f** IL-P-8. **g** IL-P-10. **h** IL-P-20.

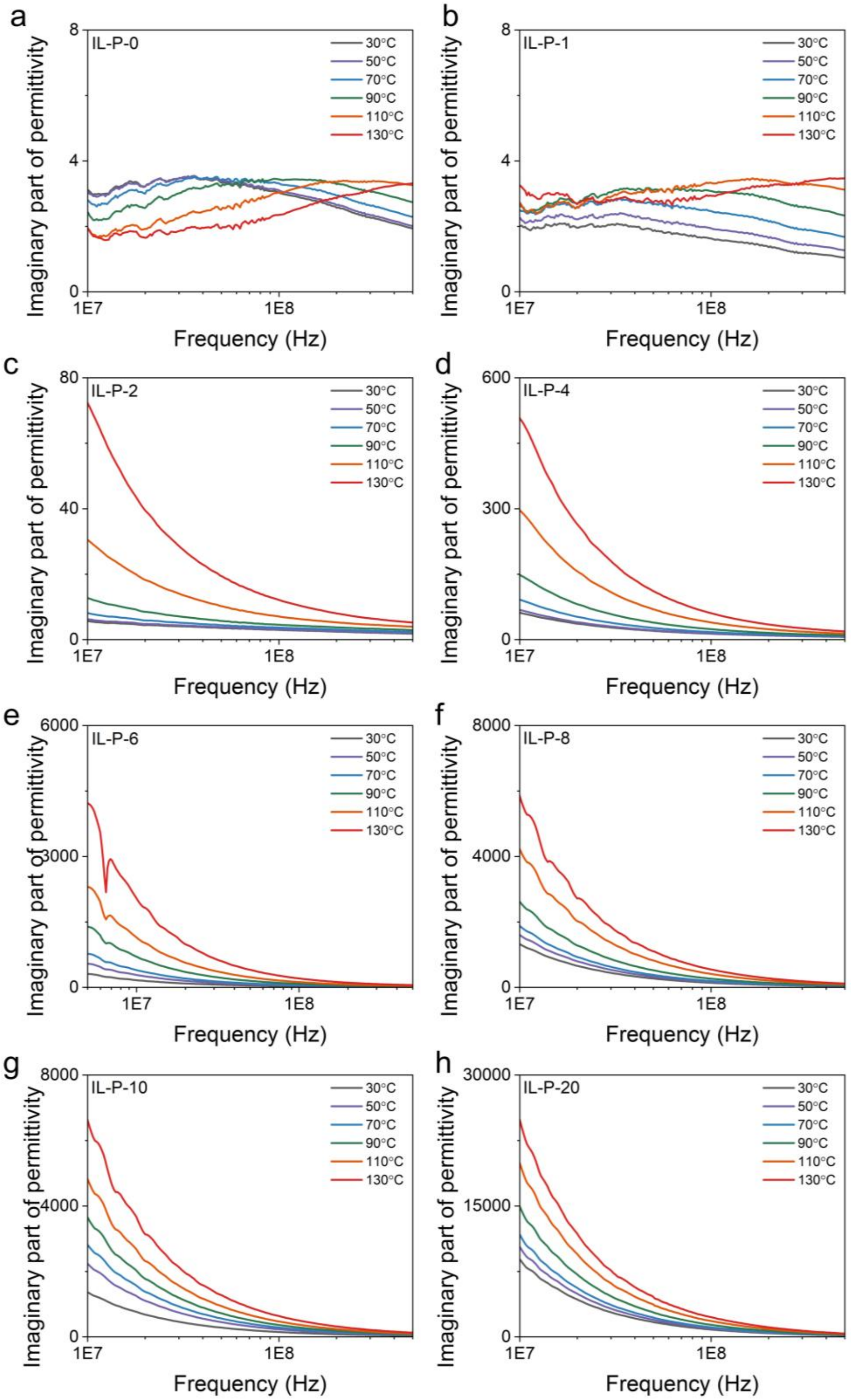

Supplementary Fig. 8 | Imaginary part of permittivity of IL-P in the 10^7-5×10^8 Hz under temperature stimulation. **a** IL-P-0. **b** IL-P-1. **c** IL-P-2. **d** IL-P-4. **e** IL-P-6. **f** IL-P-8. **g** IL-P-10. **h** IL-P-20.

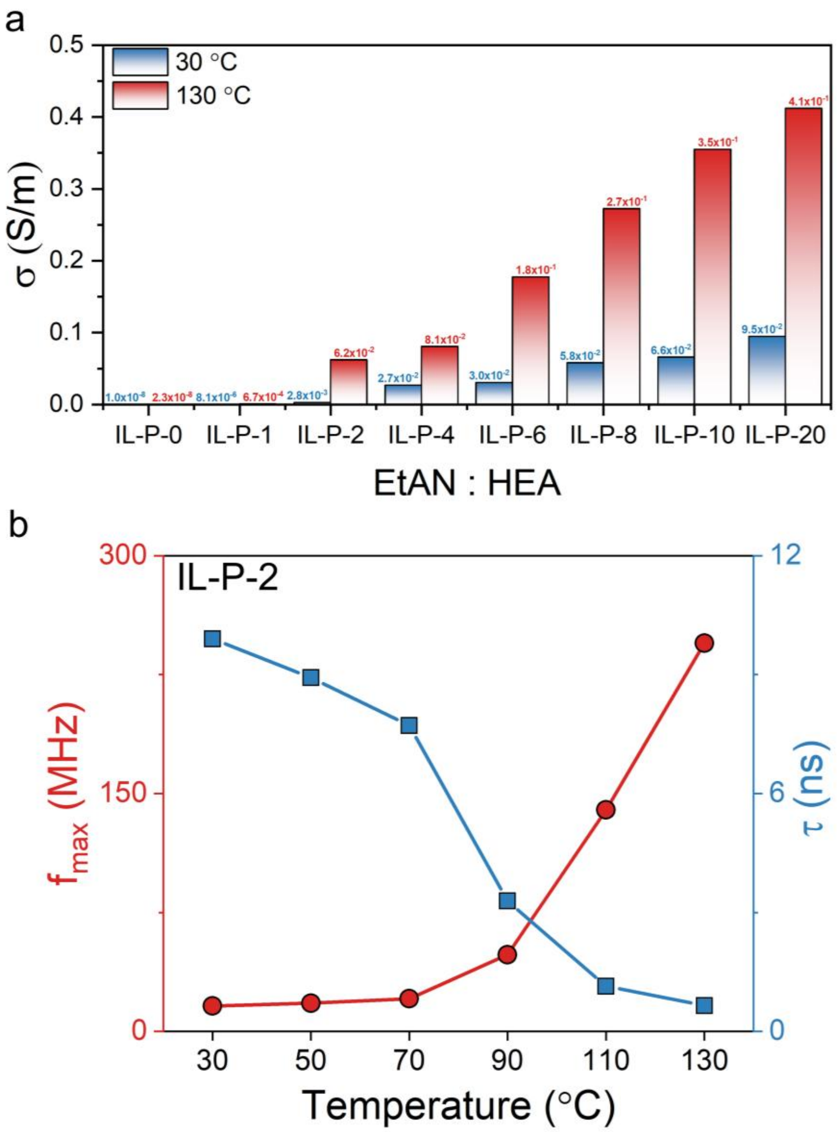

Supplementary Fig. 9 | Conductivity of IL-P and polarization relaxation of PHEA under temperature stimulation. a Comparison of the conductivity of IL-P with different concentrations at 30°C and 130°C. **b** Temperature-dependent changes in the polarization relaxation frequency and time of PHEA in the IL-P system.

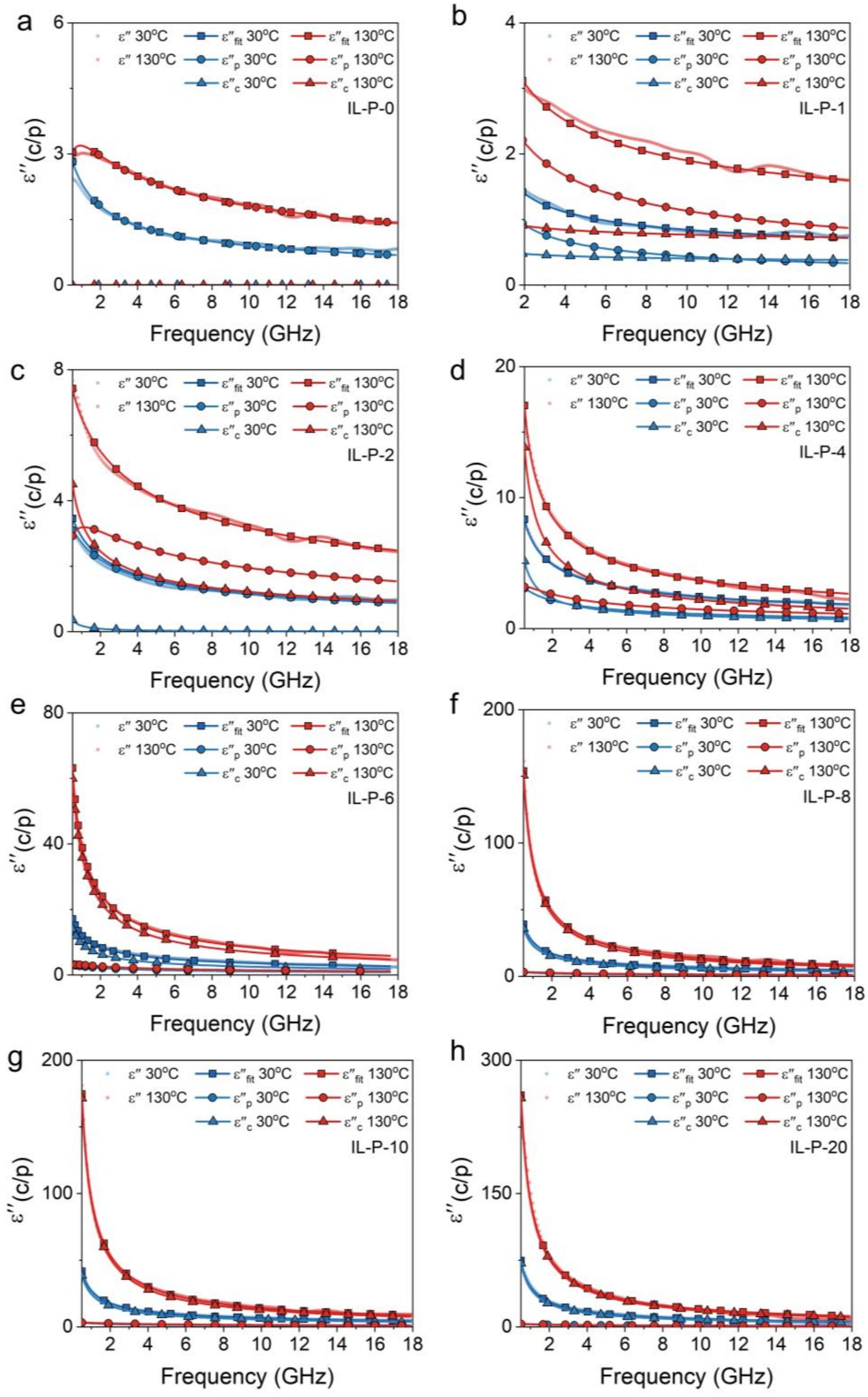

Supplementary Fig. 10 | ε''_c and ε''_p of IL-P in the 0.5–18 GHz range at 30°C and 130°C fitted using the Havriliak-Negami model. a IL-P-0. **b** IL-P-1. **c** IL-P-2. **d** IL-P-4. **e** IL-P-6. **f** IL-P-8. **g** IL-P-10. **h** IL-P-20.

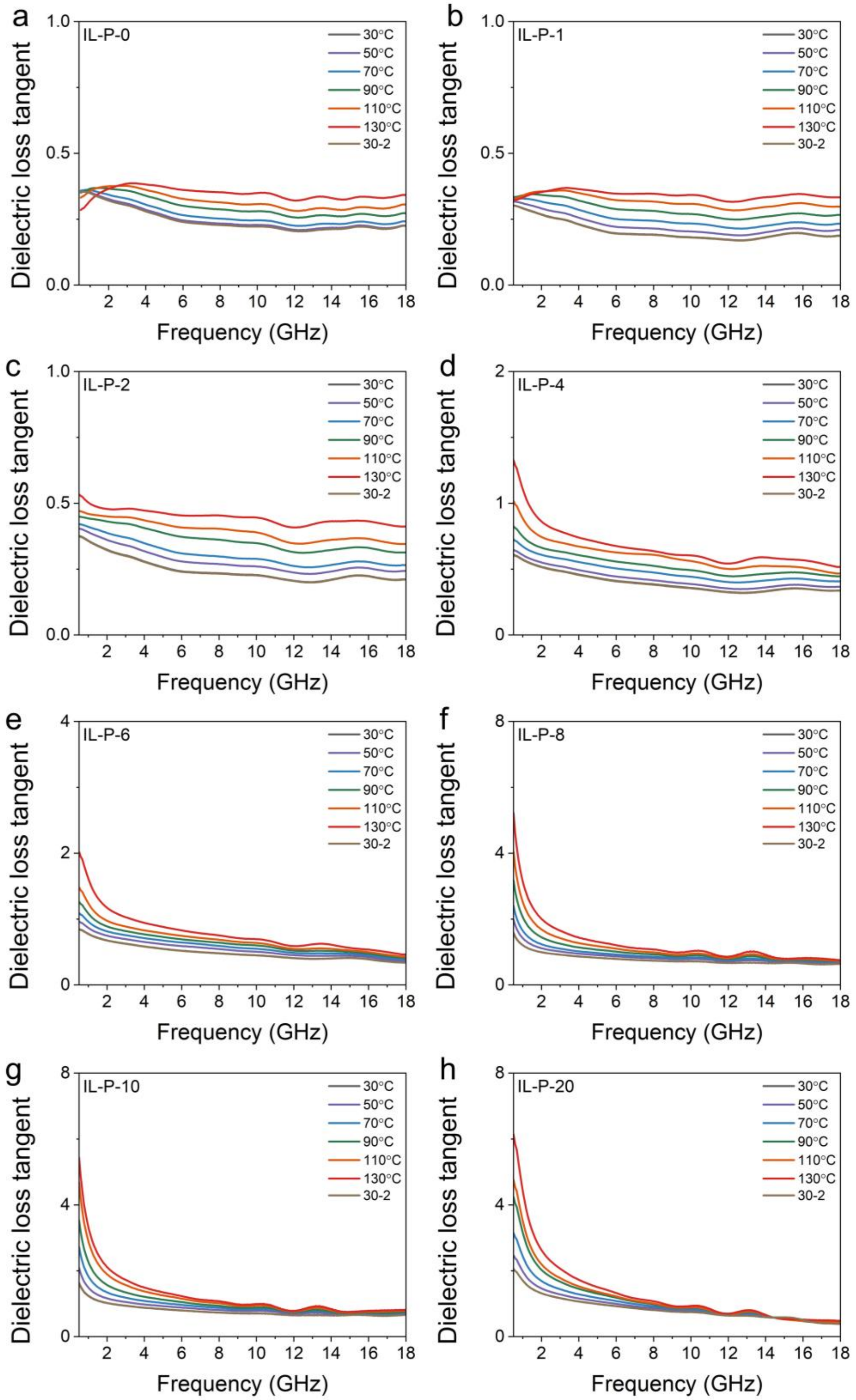

Supplementary Fig. 11 | Dielectric loss tangent of IL-P in the 0.5–18 GHz under temperature stimulation. **a** IL-P-0. **b** IL-P-1. **c** IL-P-2. **d** IL-P-4. **e** IL-P-6. **f** IL-P-8. **g** IL-P-10. **h** IL-P-20.

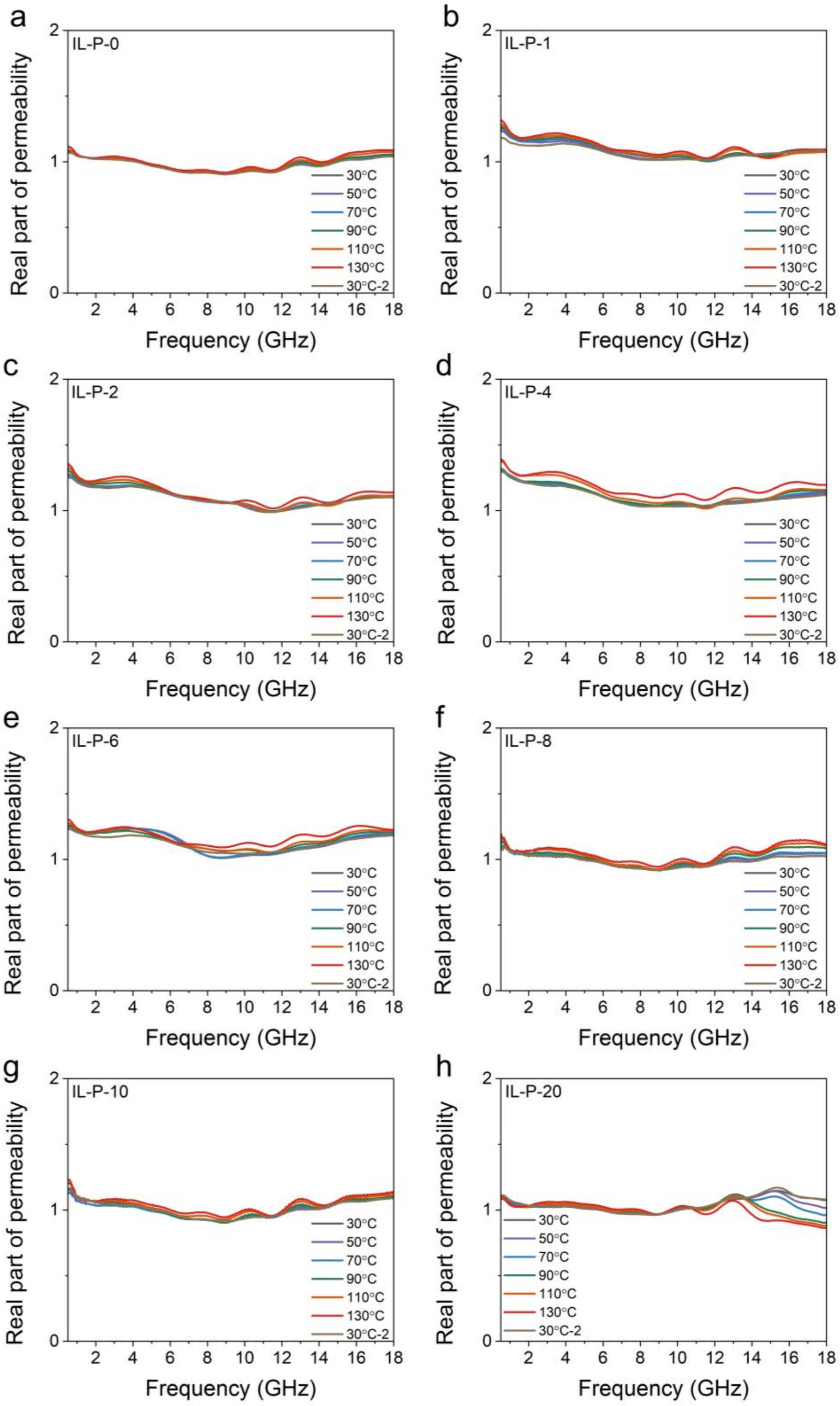

Supplementary Fig. 12 | Real part of permeability of IL-P in the 0.5–18 GHz under temperature stimulation. **a** IL-P-0. **b** IL-P-1. **c** IL-P-2. **d** IL-P-4. **e** IL-P-6. **f** IL-P-8. **g** IL-P-10. **h** IL-P-20.

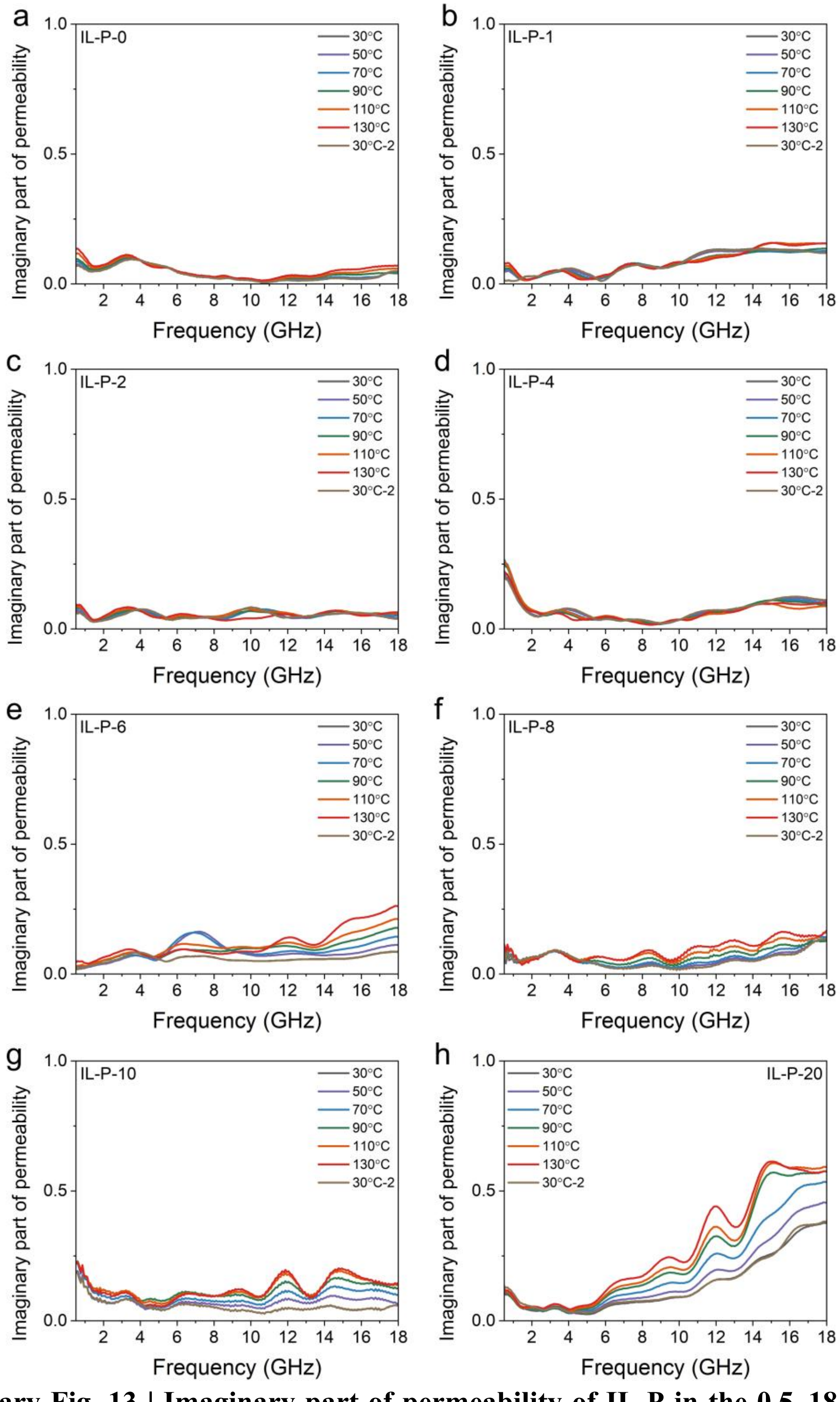

Supplementary Fig. 13 | Imaginary part of permeability of IL-P in the 0.5–18 GHz under temperature stimulation. **a** IL-P-0. **b** IL-P-1. **c** IL-P-2. **d** IL-P-4. **e** IL-P-6. **f** IL-P-8. **g** IL-P-10. **h** IL-P-20.

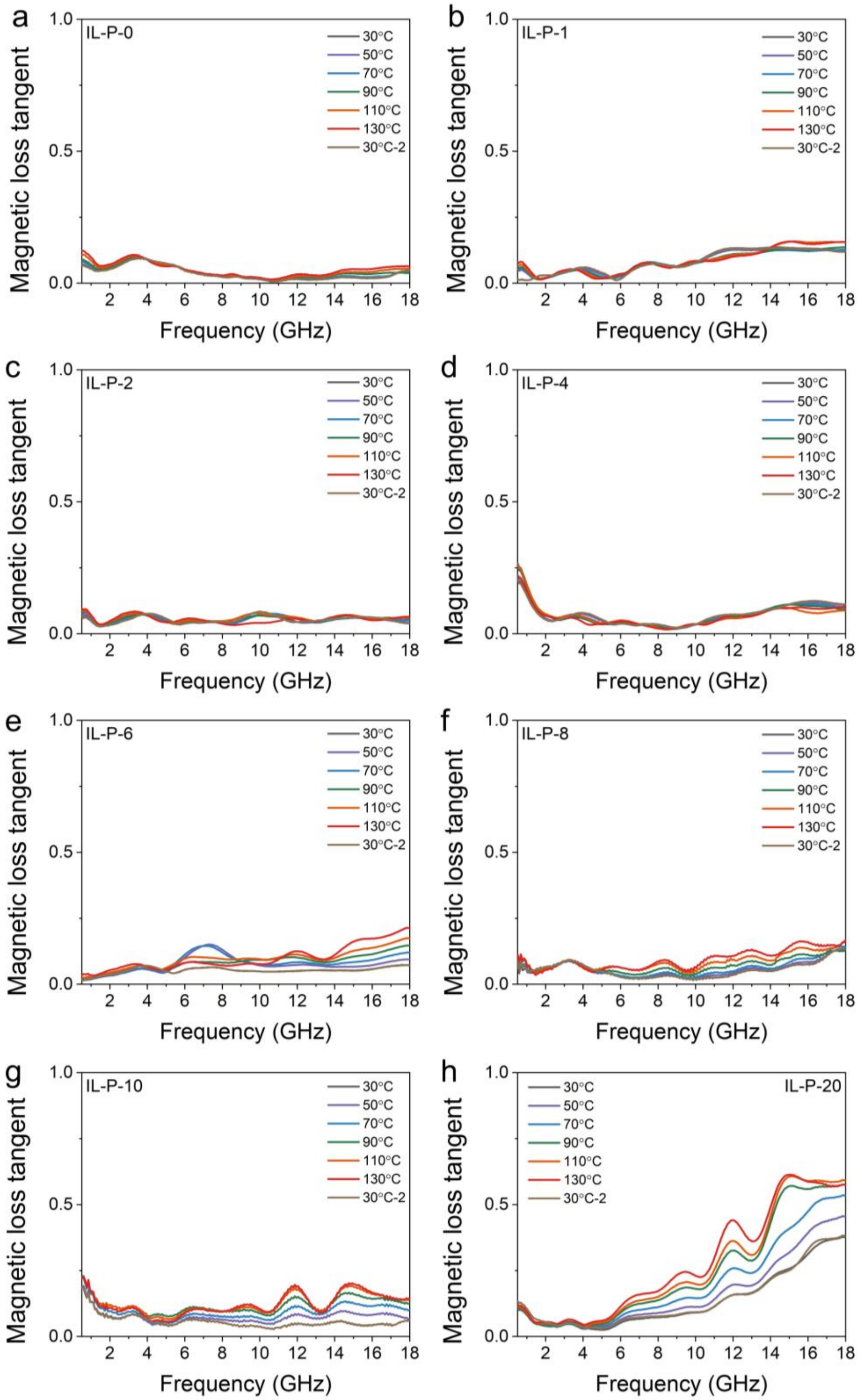

Supplementary Fig. 14 | Magnetic loss tangent of IL-P in the 0.5–18 GHz under temperature stimulation. **a** IL-P-0. **b** IL-P-1. **c** IL-P-2. **d** IL-P-4. **e** IL-P-6. **f** IL-P-8. **g** IL-P-10. **h** IL-P-20.

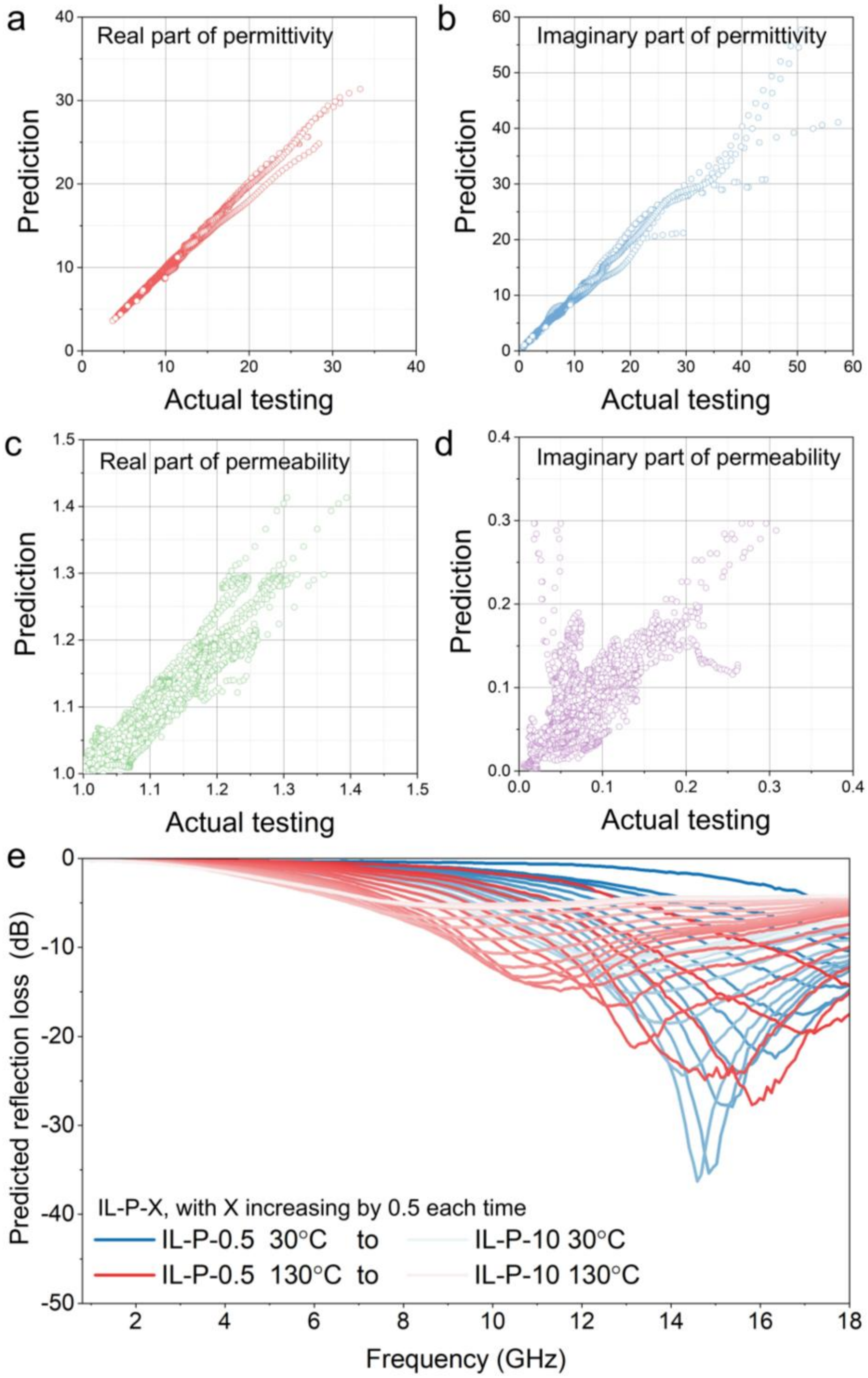

Supplementary Fig. 15 | Machine learning-based prediction results of electromagnetic parameters and reflection loss from 0.5 GHz to 18 GHz. **a** Real part of permittivity. **b** Imaginary part of permittivity. **c** Real part of the permeability. **d** Imaginary part of the permeability. **e** Reflection loss.

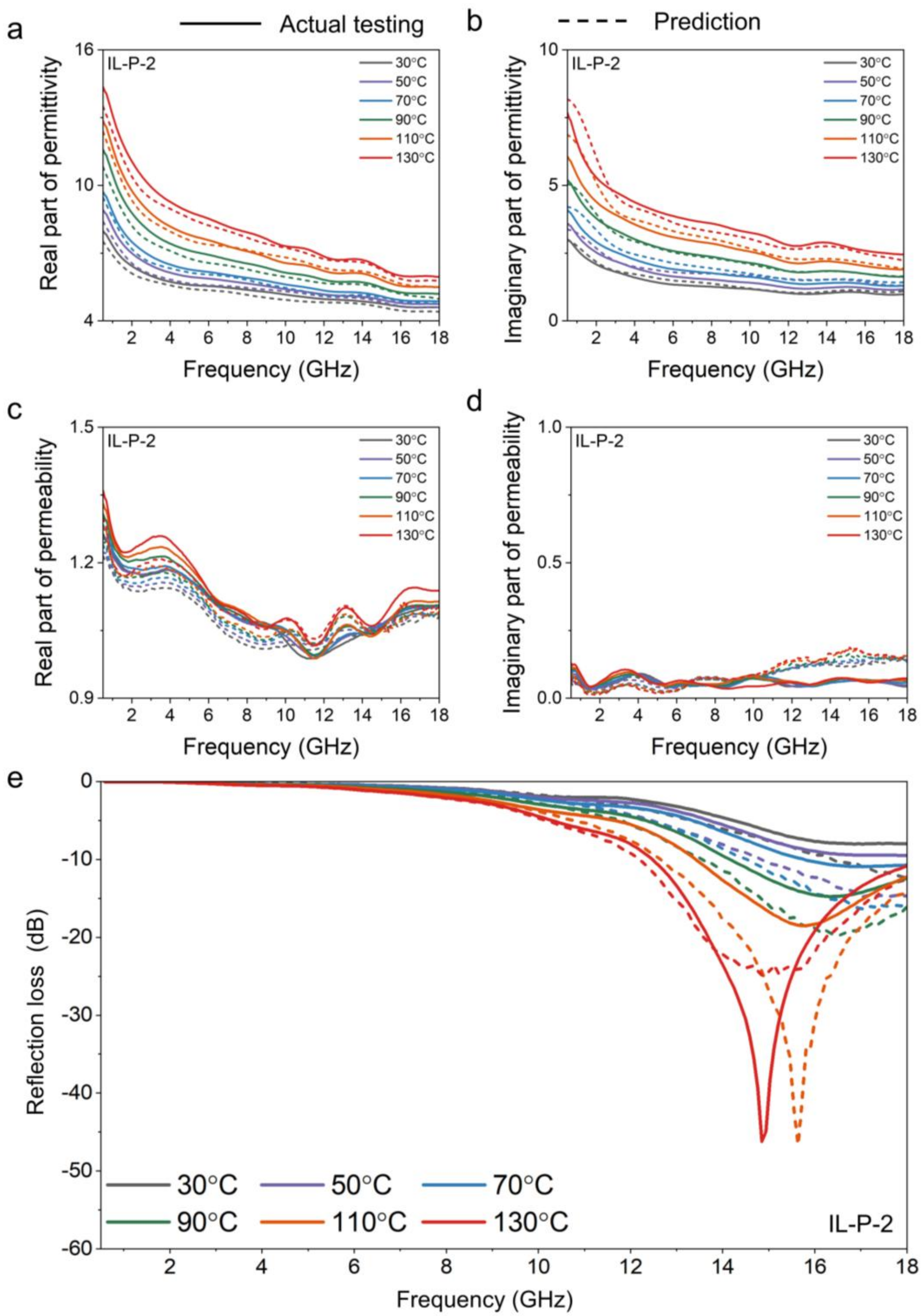

Supplementary Fig. 16 | Comparison of predicted and actual results of electromagnetic parameters and reflection loss of IL-P-2 from 0.5-18 GHz at different temperatures. a Real part of permittivity. **b** Imaginary part of permittivity. **c** Real part of the permeability. **d** Imaginary part of the permeability. **e** Reflection loss (dashed lines: predicted values, solid lines: actual values).

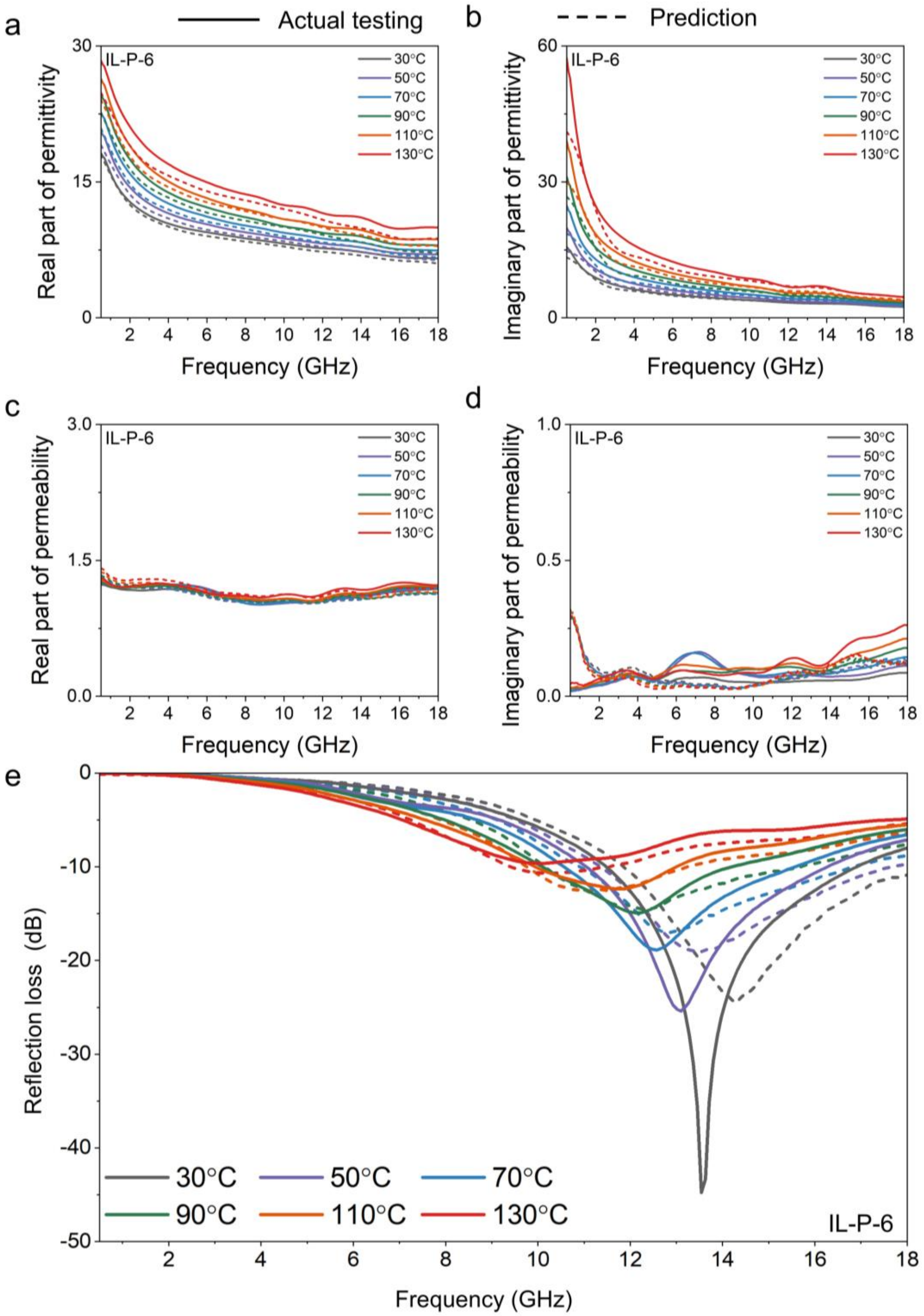

Supplementary Fig. 17 | Comparison of predicted and actual results of electromagnetic parameters and reflection loss of IL-P-6 from 0.5-18 GHz at different temperatures. a Real part of permittivity. **b** Imaginary part of permittivity. **c** Real part of the permeability. **d** Imaginary part of the permeability. **e** Reflection loss (dashed lines: predicted values, solid lines: actual values).

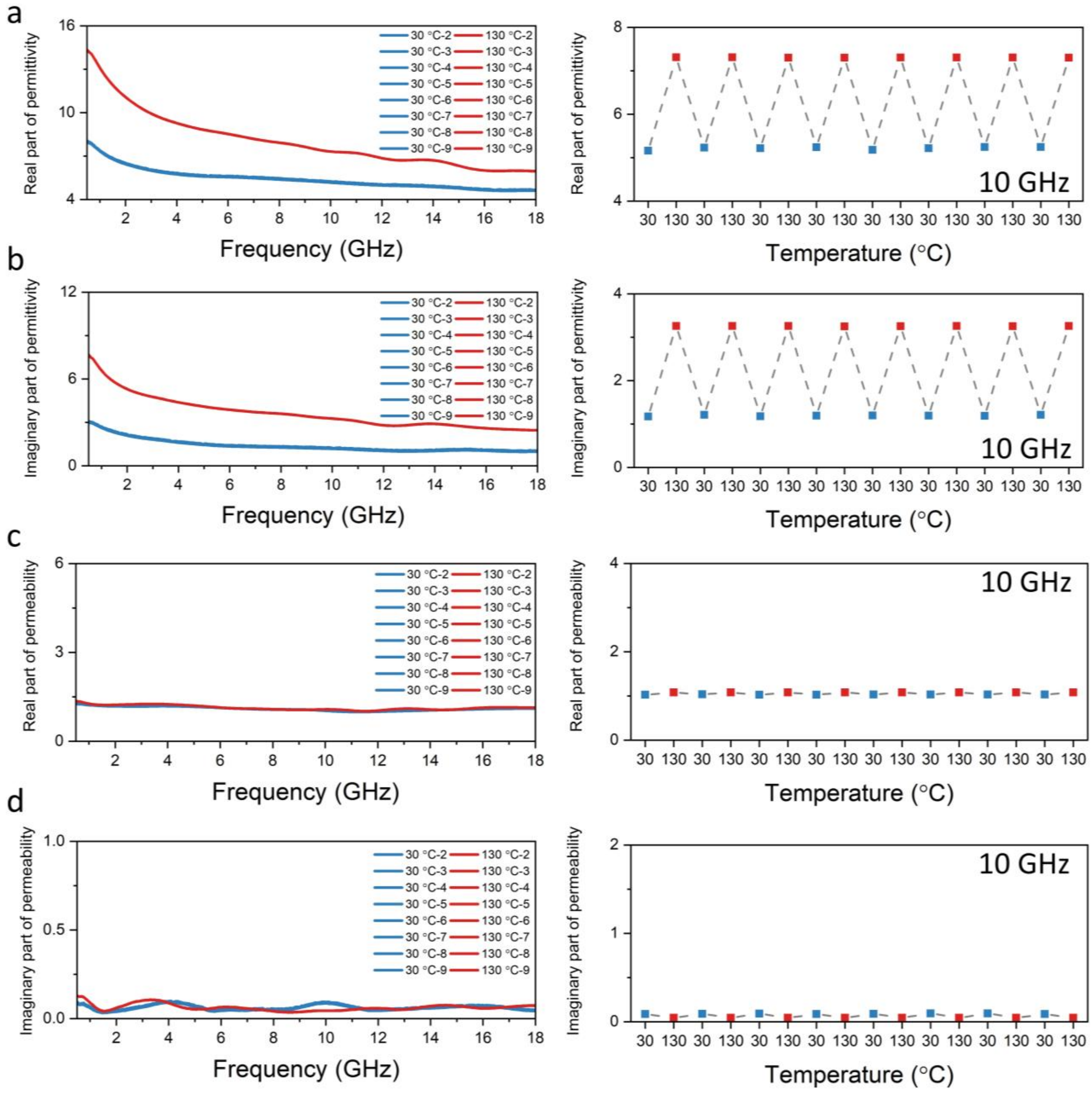

Supplementary Fig. 18 | The electromagnetic parameter cycling repeatability test for IL-P-2. a Real part of permittivity. **b** Imaginary part of permittivity. **c** Real part of the permeability. **d** Imaginary part of the permeability.

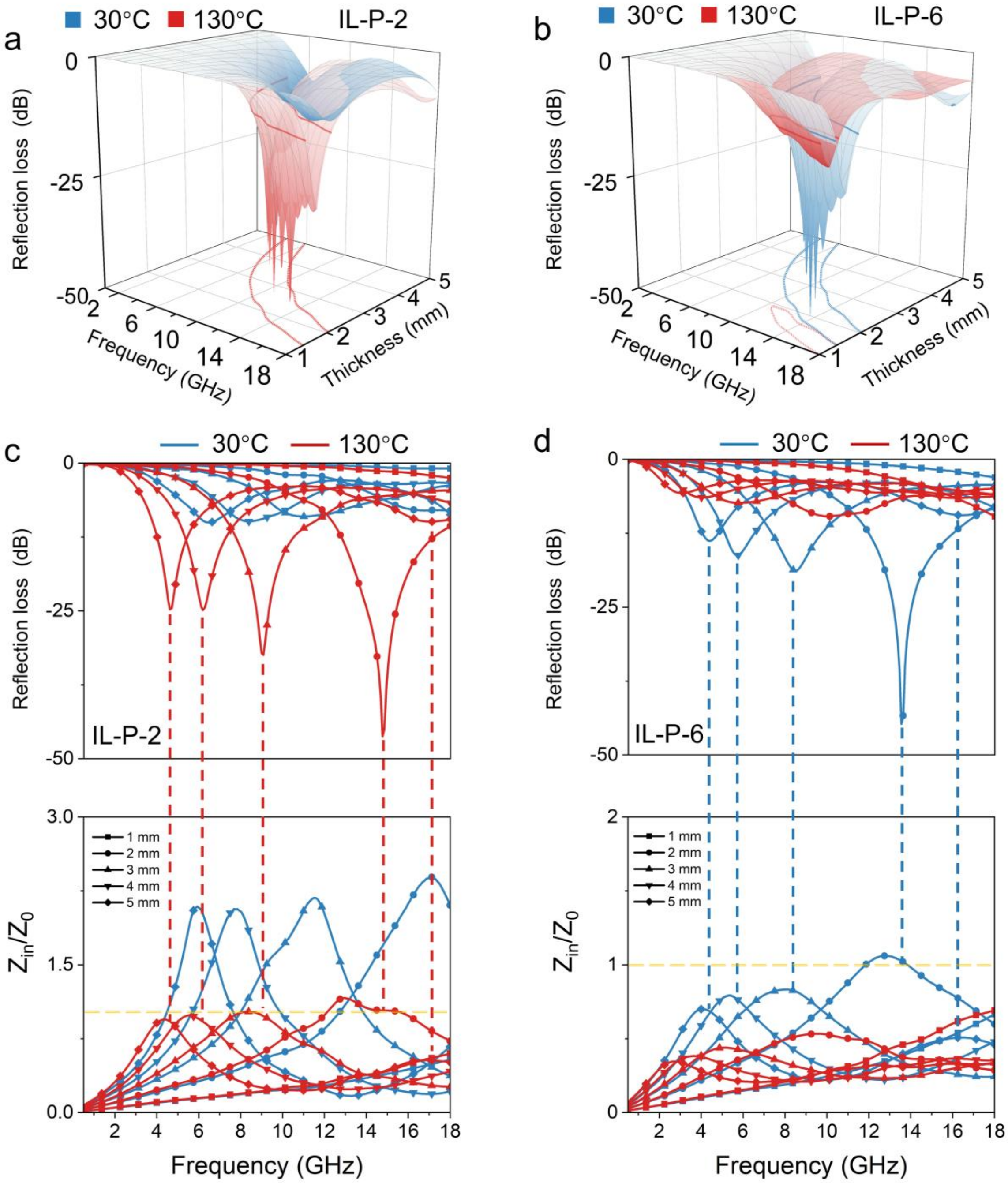

Supplementary Fig. 19 | Effect of IL-P thickness on reflection loss. a Reflection loss of IL-P-2 and IL-P-6 at different thicknesses in the 0.5–18 GHz range under temperature stimulation. **b** Z_{in}/Z_0 and corresponding reflection loss of IL-P-2 and IL-P-6 at different thicknesses at 30°C and 130°C.

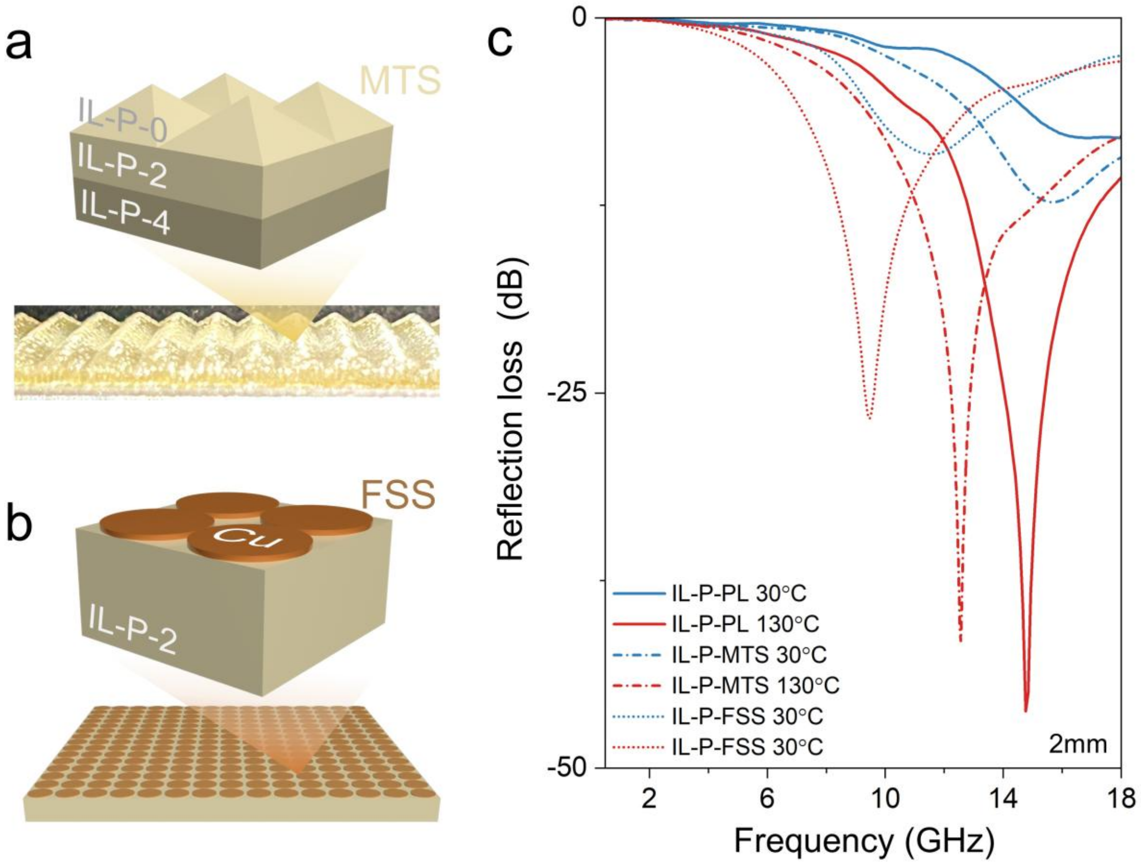

Supplementary Fig. 20 | Microwave Modulation Performance of IL-P and Metasurfaces. a Multilayer photopolymerized 3D-printed IL-P-MTS metamaterial structure. **b** IL-P-FSS structure is formed by combining a metallic disk array with IL-P. **c** RL of the metasurface IL-P in the 0.5–18 GHz.

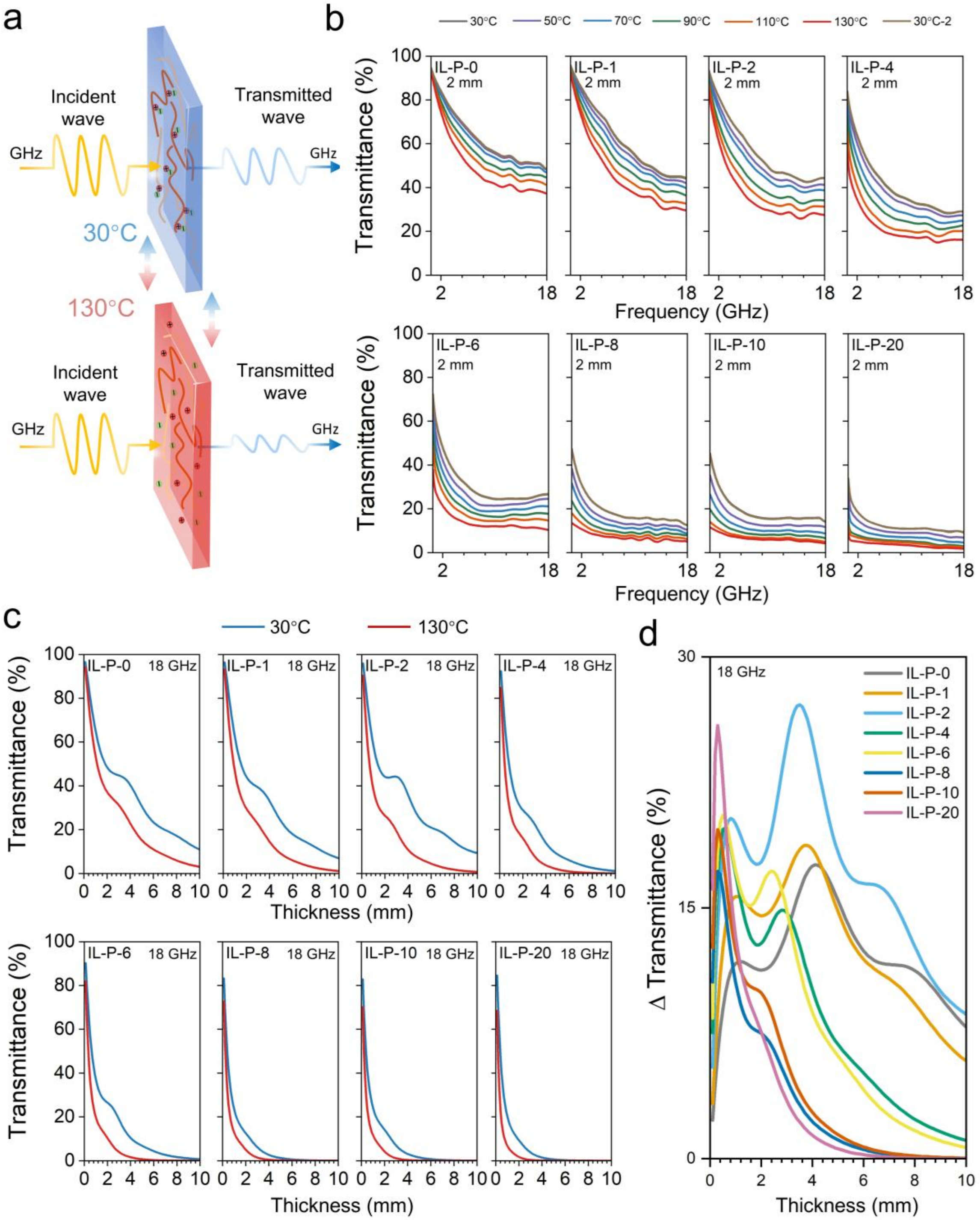

Supplementary Fig. 21 | Microwave transmission Properties of IL-P Active Surfaces. a Schematic illustration of tunable transmission in IL-P. **b** Transmission rate of 2 mm-thick IL-P in the 0.5–18 GHz range under different temperature stimuli. **c** Transmission rate of IL-P with different thicknesses at 18 GHz at 30 °C and 130 °C. **d** Modulation amplitude of transmission rate for IL-P with different thicknesses at 18 GHz.

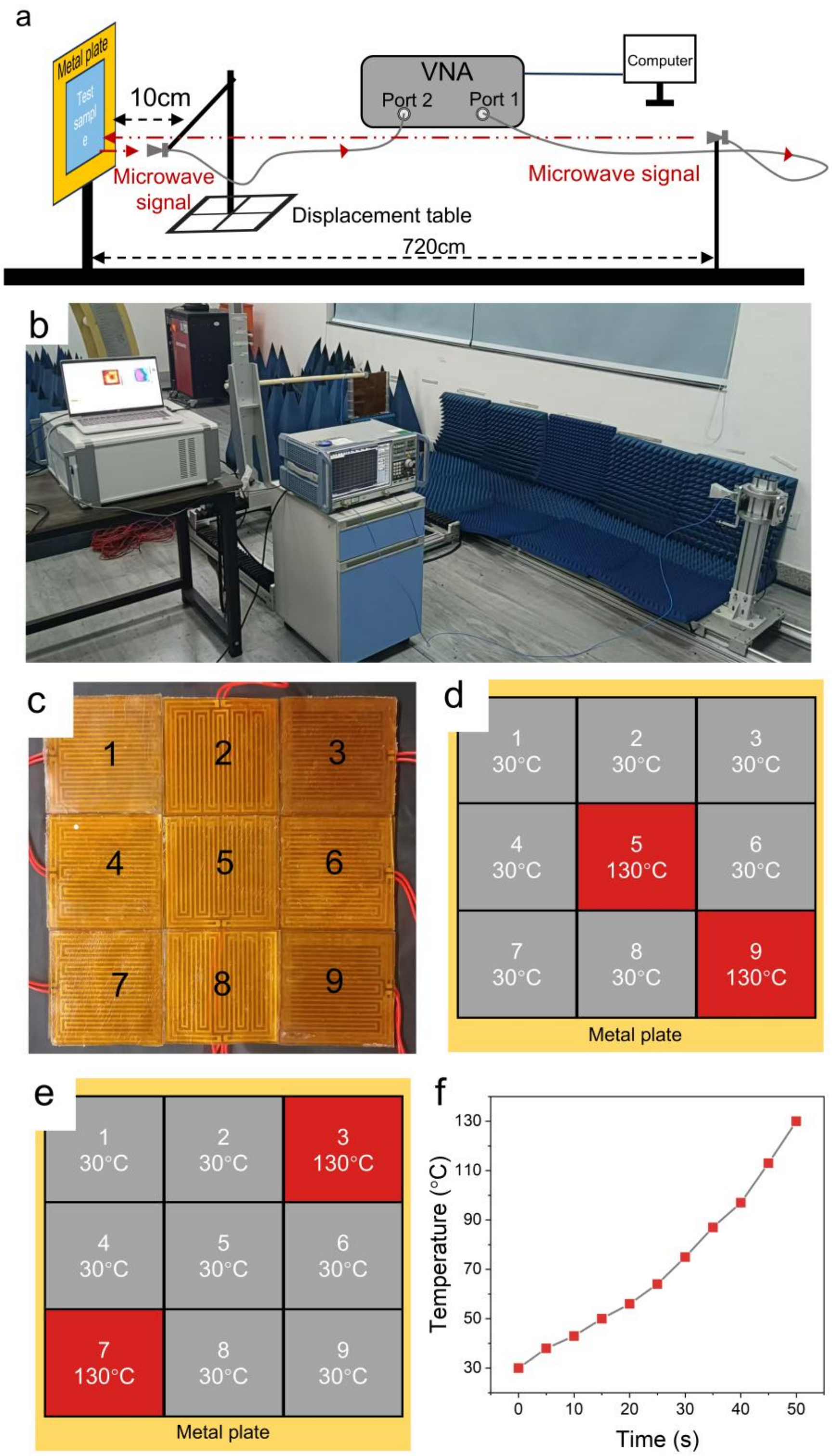

Supplementary Fig. 22 | Pixelated microwave absorbing surface imaging. a Schematic of far-field imaging test. **b** Far-field imaging system. **c** Far-field imaging model. **d, e** Schematic of pixelated imaging control under temperature stimulation. **f** The temperature rise rate of the microwave absorbing surface with a bottom heating plate at 24V.

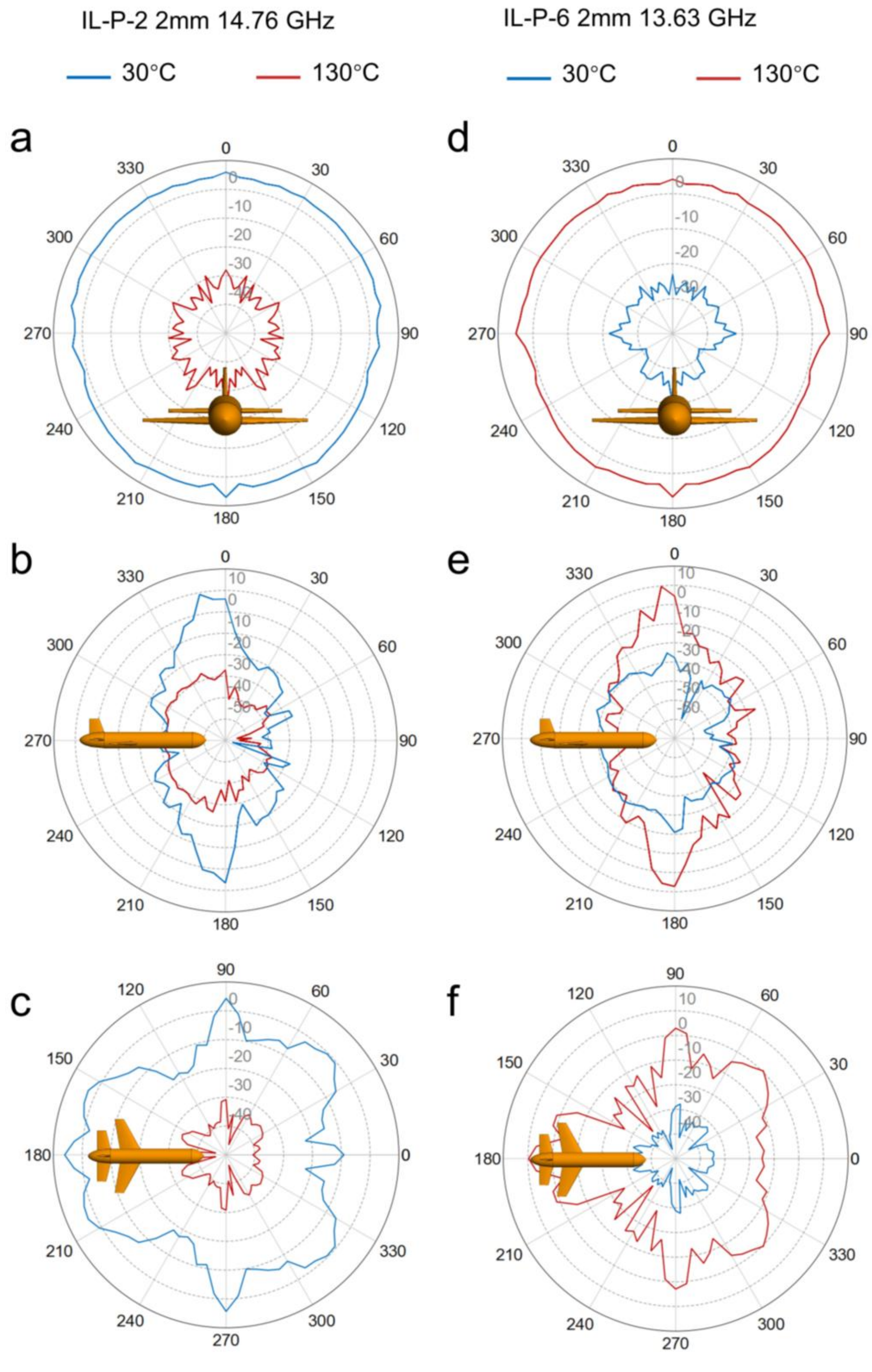

Supplementary Fig. 23 | Switchable RCS on the aircraft surface. a-c Simulated RCS (dBsm) results of 2 mm-thick IL-P-2 at different cross-sections at 30°C and 130°C. **d-f** Simulated RCS (dBsm) results of 2 mm-thick IL-P-6 at different cross-sections at 30°C and 130°C.

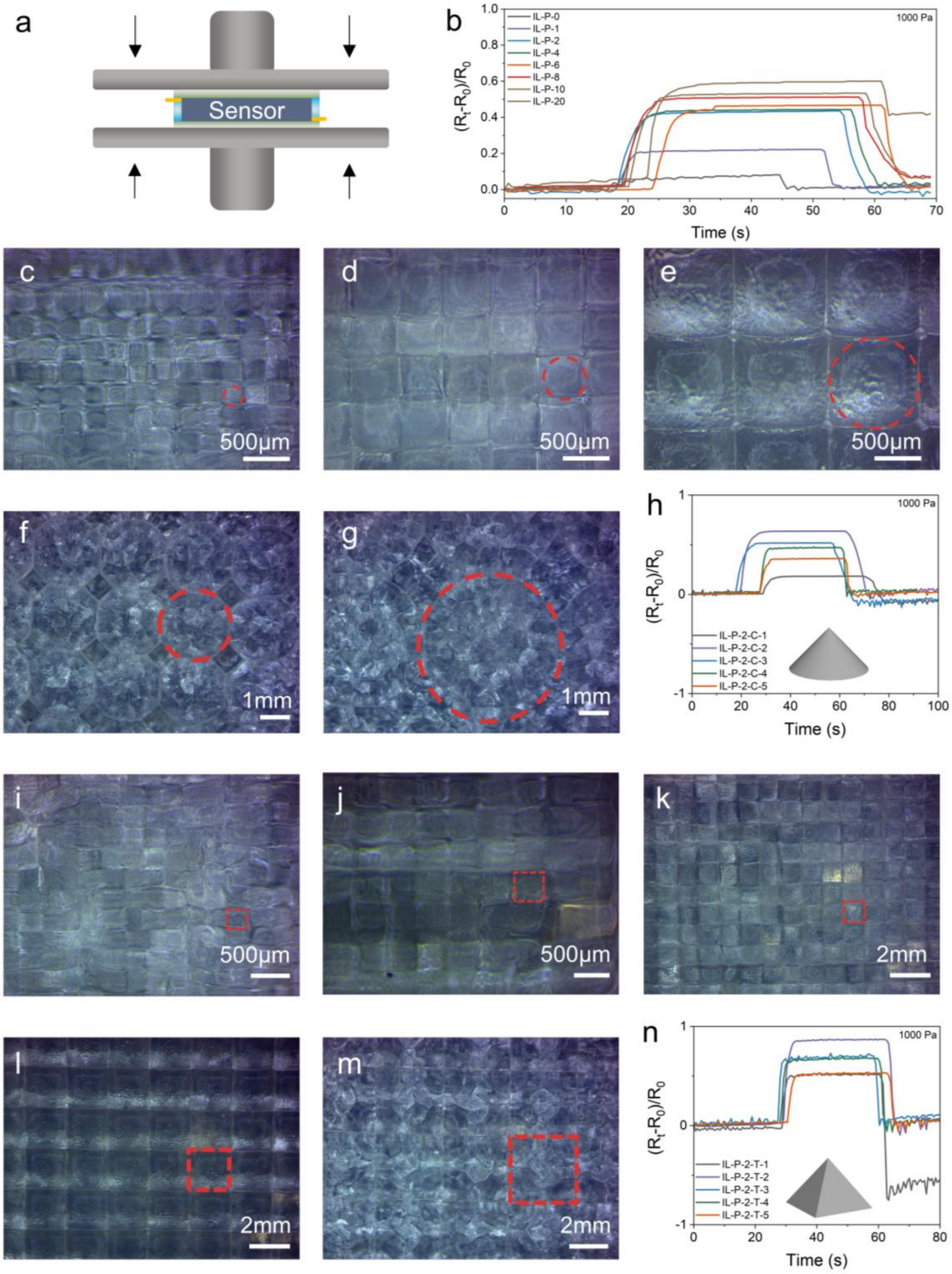

Supplementary Fig. 24 | IL-P capacitive mechanical sensors. a Schematic of IL-P sensor module testing. **b** Dynamic mechanical sensing performance of IL-P sensing materials with different IL concentrations and 500 μm thickness. **c-h** Microscopic morphology and sensing performance of IL-P-2-C with different radii of the conical shape. **i-n** Microscopic morphology and sensing performance of IL-P-2-T with different lengths of the truncated conical shape.

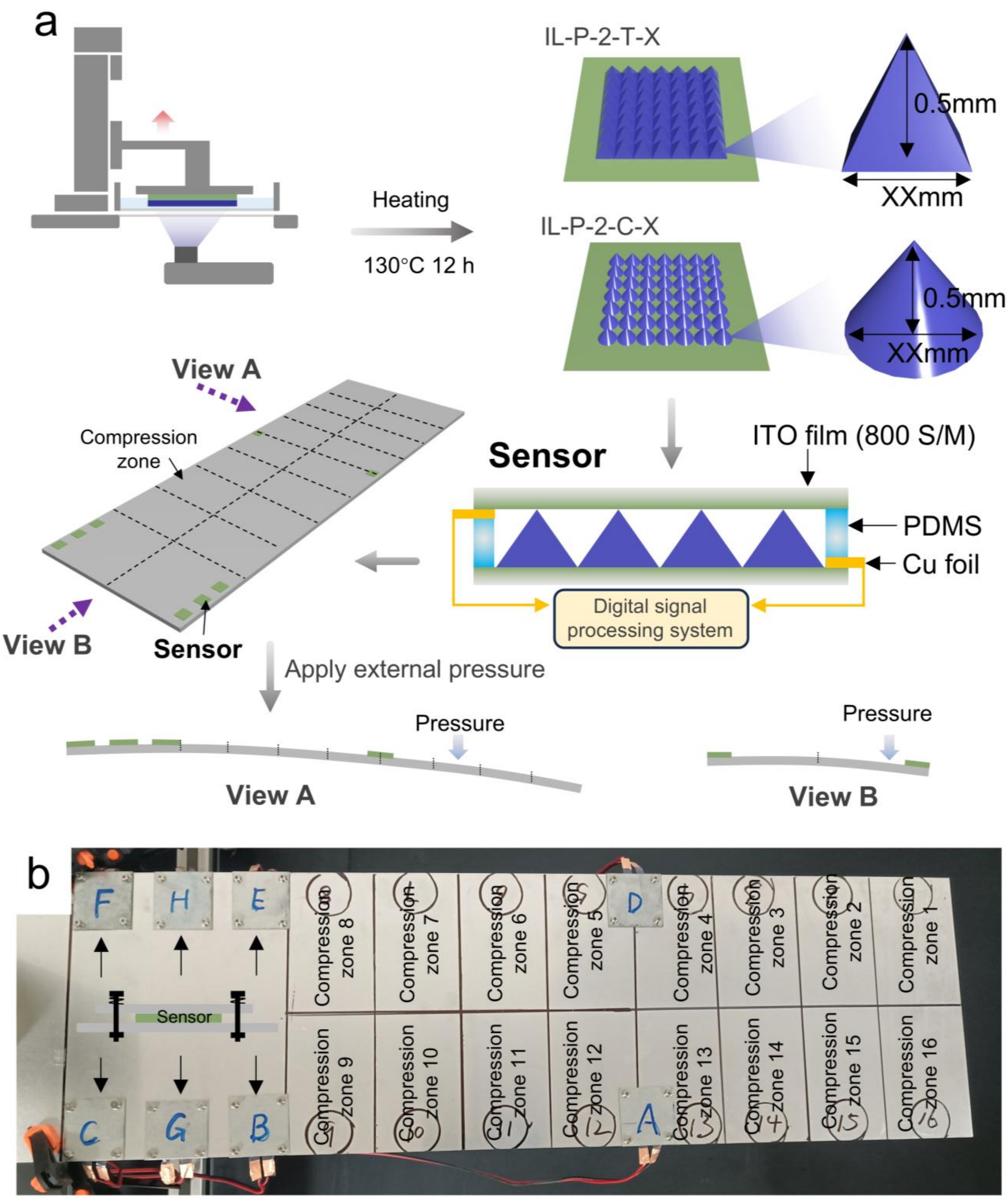

Supplementary Fig. 25 | Self-sensing surface of IL-P. a Schematic of IL-P sensor module and self-sensing surface fabrication. **b** Self-sensing surface composed of IL-P sensor array.

Supplementary Tab. 1 Comparison of IL-P with other tunable dielectric surfaces.

Type	Material	State	Frequency	ε′ (10GHz)	ε″ (10GHz)	Method	Mechanism	Ref.
Liquid Crystal Metasurfaces	E7	Liquid	Light 500-900 nm	_	_	Temperature	Molecular arrangement	10
Liquid Crystal Metasurfaces	E7	Liquid	Light 580-700 nm	_	_	Voltage	Molecular arrangement	11
Liquid Crystal Metasurfaces	E7	Liquid	Light 1500-1750 nm	_	_	Voltage	Molecular arrangement	12
Active metallic metasurface	Varicap diode	Solid	Microwave 2.6-4.0 GHz	_	_	Voltage	Reconfigurable metasurfaces	13
Active metallic metasurface	Varicap diode	Solid	Microwave 8.4 GHz	_	_	Voltage	Reconfigurable metasurfaces	14
Active metallic metasurface	Varicap diode	Solid	Microwave 6-22 GHz	_	_	Voltage	Reconfigurable metasurfaces	15
Dynamic metasurface	Dynamic array	Solid	Microwave 4-12 GHz	_	_	Deformation	Reconfigurable metasurfaces	16
Metal-oxide composit foam	RGO/VO_2 Aerogel	Solid	Microwave 1-18 GHz	10	3 to 5	Temperature	Conductivity	17
Ionic liquid polymer	IL-P-2<br>IL-P-6	Solid	Microwave 2-18 GHz	5.1 to 7.3<br>8.1 to 12.4	1.1 to 3.2<br>3.6 to 8.6	Temperature	Primary: Ionic conductivity<br>Secondary: Molecular orientation movement	This work

References

1 Zhang, X. et al. Metal-organic frameworks with fine-tuned interlayer spacing for microwave absorption. *Sci. Adv.* **10**, 6498 (2024).

2 Grosse, C. A program for the fitting of up to three Havriliak-Negami dispersions to dielectric data. *J. Colloid Interface Sci.* **600**, 318-323 (2021).

3 Zhang, Y. et al. An Urchin-Inspired Broadband and Ultralight Microwave Absorber. *Adv. Funct. Mater.* **35**, 2419943 (2025).

4 Gong, X. et al. Trunk-Inspired SWCNT-Based Wrinkled Films for Highly-Stretchable Electromagnetic Interference Shielding and Wearable Thermotherapy. *Nano-Micro. Lett.* **16**, 243 (2024).

5 Yan, H. et al. Dielectric–magnetic synergistic design of Ti_3C_2Tx@C/NiZn ferrite composite for effective microwave absorption performance. *Appl. Surf. Sci.* **633**, 157602 (2023).

6 Tian, W. et al. Atomic-Scale Layer-by-Layer Deposition of FeSiAl@ZnO@Al_2O_3 Hybrid with Threshold Anti-Corrosion and Ultra-High Microwave Absorption Properties in Low-Frequency Bands. *Nano-Micro. Lett.* **13**, 161 (2021).

7 Dai, Y., Wu, X., Liu, Z., Zhang, H. B. & Yu, Z. Z. Highly sensitive, robust and anisotropic MXene aerogels for efficient broadband microwave absorption. *Compos. Part B-Eng.* **200**, 108263 (2020).

8 Cheng, Z. et al. Intelligent Off/On Switchable Microwave Absorption Performance of Reduced Graphene Oxide/VO_2 Composite Aerogel. *Adv. Funct. Mater.* **32**, 2205160 (2022).

9 Duan, L. et al. Electron Migratory Polarization of Interfacial Electric Fields Facilitates Efficient Microwave Absorption. *Adv. Funct. Mater.* **35**, 2416727 (2025).

10 Komar, A. et al. Dynamic Beam Switching by Liquid Crystal Tunable Dielectric Metasurfaces. *ACS Photonics* **5**, 1742-1748 (2018).

11 Zou, C. et al. Electrically Tunable Transparent Displays for Visible Light Based on Dielectric Metasurfaces. *ACS Photonics* **6**, 1533-1540 (2019).

12 Komar, A. et al. Electrically tunable all-dielectric optical metasurfaces based on liquid crystals. *Appl. Phys. Lett.* **110**, 071109 (2017).

13 Li, F. et al. Flexible intelligent microwave metasurface with shape-guided adaptive programming. *Nat. Commun.* **16**, 3161 (2025).

14 Qian, C. et al. Deep-learning-enabled self-adaptive microwave cloak without human intervention. *Nat. Photonics* **14**, 383-390 (2020).

15 Li, W., Xu, M., Xu, H. X., Wang, X. & Huang, W. Metamaterial Absorbers: From Tunable Surface to Structural Transformation. *Adv. Mater.* **34**, 2202509 (2022).

16 Lim, D. D. et al. A tunable metamaterial microwave absorber inspired by chameleon's color-changing mechanism. *Sci. Adv.* **11**, 3499 (2025).

17 Cheng, Z. et al. Intelligent Off/On Switchable Microwave Absorption Performance of Reduced Graphene Oxide/VO_2 Composite Aerogel. *Adv. Funct. Mater.* **32**, 2205160 (2022).
